# Role of the Microbiome in the Pathogenesis of COVID-19

**DOI:** 10.3389/fcimb.2022.736397

**Published:** 2022-03-31

**Authors:** Rituparna De, Shanta Dutta

**Affiliations:** ^1^ Division of Bacteriology, National Institute of Cholera and Enteric Diseases, Kolkota, India; ^2^ Division of Immunology, National Institute of Cholera and Enteric Diseases, Kolkota, India

**Keywords:** COVID-19, microbiome, SARS-CoV2, inflammation, ACE2, serotonin, kynurenine

## Abstract

The ongoing pandemic coronavirus disease COVID-19 is caused by the highly contagious single-stranded RNA virus, SARS-coronavirus 2 (SARS-CoV-2), which has a high rate of evolution like other RNA viruses. The first genome sequences of SARS-CoV-2 were available in early 2020. Subsequent whole-genome sequencing revealed that the virus had accumulated several mutations in genes associated with viral replication and pathogenesis. These variants showed enhanced transmissibility and infectivity. Soon after the first outbreak due to the wild-type strain in December 2019, a genetic variant D614G emerged in late January to early February 2020 and became the dominant genotype worldwide. Thereafter, several variants emerged, which were found to harbor mutations in essential viral genes encoding proteins that could act as drug and vaccine targets. Numerous vaccines have been successfully developed to assuage the burden of COVID-19. These have different rates of efficacy, including, although rarely, a number of vaccinated individuals exhibiting side effects like thrombosis. However, the recent emergence of the Britain strain with 70% more transmissibility and South African variants with higher resistance to vaccines at a time when several countries have approved these for mass immunization has raised tremendous concern regarding the long-lasting impact of currently available prophylaxis. Apart from studies addressing the pathophysiology, pathogenesis, and therapeutic targets of SARS-CoV-2, analysis of the gut, oral, nasopharyngeal, and lung microbiome dysbiosis has also been undertaken to find a link between the microbiome and the pathogenesis of COVID-19. Therefore, in the current scenario of skepticism regarding vaccine efficacy and challenges over the direct effects of currently available drugs looming large, investigation of alternative therapeutic avenues based on the microbiome can be a rewarding finding. This review presents the currently available understanding of microbiome dysbiosis and its association with cause and consequence of COVID-19. Taking cues from other inflammatory diseases, we propose a hypothesis of how the microbiome may be influencing homeostasis, pro-inflammatory condition, and the onset of inflammation. This accentuates the importance of a healthy microbiome as a protective element to prevent the onset of COVID-19. Finally, the review attempts to identify areas where the application of microbiome research can help in reducing the burden of the disease.

## Introduction

SARS-CoV-2 (severe acute respiratory syndrome coronavirus 2) is the causative agent of the novel coronavirus disease COVID-19, one of the worst pandemics in documented history (Morens et al., 2020). Individual SARS-CoV-2 is classified under realm *Riboviria* and order *Nidovirales*, suborder *Cornidovirineae*, family *Coronaviridae*, subfamily Orthocoronavirinae, genus *Betacoronavirus*, subgenus *Sarbecovirus*, and species severe acute respiratory syndrome-related coronavirus (Coronaviridae Study Group of the International Committee on Taxonomy of Viruses, 2020). It is of zoonotic origin, and transmission occurs by droplets and surface contact (Tay et al., 2020). The air-borne transmission theory of the virus has been widely debated (Greenhalgh et al., 2021; Samet et al., 2021). Indirect evidence suggests that the air-borne transmission hypothesis may be true (Greenhalgh et al., 2021; Samet et al., 2021). Other possible routes of transmission are aerosol (Bchetnia et al., 2020; Salzberger et al., 2020) and the fecal–oral route (Tay et al., 2020; Walsh et al., 2020; Jiao et al., 2021). COVID-19 is a highly contagious disease and spreads rapidly (has a reproductive number R0 in the range 2–3) (Li et al., 2020). It affects the lower respiratory tract causing severe pneumonia and acute respiratory distress syndrome (ARDS) and multiorgan failure in susceptible individuals leading to death in the most severe cases (Bchetnia et al., 2020; Tay et al., 2020). The individuals mostly at risk are those with weakened and dysregulated immune system and those with comorbidities like cardiovascular disease, type 2 diabetes, hypercholesterolemia, chronic obstructive pulmonary disease, hypertension, asthma, cancer, dementia, obesity, and other underlying clinical conditions (Dolk, 2020; Grasselli et al., 2020; Leon et al., 2020). Other risk groups include immunocompromised individuals, old age population, and those undergoing surgery and organ transplantation (Williamson et al., 2020). Males compared to females and Africans and South Asians are more prone to the disease (Williamson et al., 2020). GWAS have been conducted to identify the genetic basis of COVID-19 susceptibility (Karim et al., 2020; Katz et al., 2020). Susceptibility to respiratory failure in infected patients has been found to be linked to the genetic background of the individual (Karim et al., 2020; Katz et al., 2020). Determinants of disease severity lie in host factors (Kaser, 2020). Variants at two loci have been identified to be associated with susceptibility to severe COVID-19 (Kaser, 2020). The rs657152-A variant at ABO locus 9q34.2 has been found to be responsible for deep-vein thrombosis, pulmonary embolism, and elevated levels of the von Willebrand factor and factor VIII and also high levels of interleukin-6, which are seen in patients during disease severity (Karim et al., 2020; Katz et al., 2020). Blood groups A and B are more at risk of thromboembolism than group O irrespective of the COVID-19 status (Karim et al., 2020; Katz et al., 2020). rs505922-C polymorphism has been found to be associated with higher levels of the soluble lectin CD209 (Karim et al., 2020; Katz et al., 2020).Variants at the multigene locus 3p21.31 are associated with levels of CXCL16, an inflammatory chemokine associated with alveolitis and atherogenesis (Kaser, 2020; Katz et al., 2020). An insertion deletion GA or G variant at rs11385942 in the *LZTFL1* gene at locus 3p21.31 is associated with a predisposition toward the most severe forms of COVID-19 (Kaser, 2020).

Common symptoms of COVID-19 include fever, dry cough, shortness of breath, and fatigue (Wiersinga et al., 2020). Other abnormalities like lymphopenia and elevated levels of lactate dehydrogenase, inflammatory markers like TNF-α, IL-6, IL-1, ferritin, C-reactive protein, and low levels of albumin may be observed (Wiersinga et al., 2020). Few patients may report leukocytosis and elevated levels of procalcitonin (Contini et al., 2020). Other less common symptoms are myalgia, asthenia, chills, rhinorrhea, diarrhea, nausea, headache, weakness, anosmia or ageusia, instability, ideomotor slowdown, ataxia, epilepsy, hypogeusia, hyposmia, and neuralgia (Contini et al., 2020; Wiersinga et al., 2020). Encephalopathy, encephalitis, necrotizing hemorrhagic encephalopathy, stroke, epileptic seizures, rhabdomyolysis, and Guillain–Barre syndrome have also been observed (Contini et al., 2020). The severity of these symptoms varies in an age-dependent manner as found by Contini et al. (Contini et al., 2020). About 5%–10% of patients require hospitalization (Jiang et al., 2020). In 5% of patients of COVID-19 and in 20% hospitalized patients, severe symptoms develop demanding intensive care (Jiang et al., 2020; Wiersinga et al., 2020). About 75% of these hospitalized patients require supplemental oxygen (Wiersinga et al., 2020).

Symptoms develop usually within 4–5 days and in 97.5% individuals within 11.5 days (Lauer et al., 2020; Wiersinga et al., 2020). The mean incubation period is 5 days (Wiersinga et al., 2020), the median incubation period is 5.1 days (Lauer et al., 2020), while 101 out of 10,000 cases develop symptoms after 14 days (Lauer et al., 2020). The current method of diagnosis includes RT-PCR from upper (like nasopharyngeal/oropharyngeal or nasal swabs, saliva) and lower respiratory (sputum, tracheal aspirate, BAL) samples (Murphy, 2020). Lower respiratory tract samples have shown higher sensitivity than upper respiratory tract samples for detection and diagnosis through RT-PCR (Murphy, 2020). The viral load is the highest within 5–6 days of the symptom onset (Tay et al., 2020). In severe cases, ARDS develops on average within 8–9 days after the symptom onset (Tay et al., 2020). The virus cannot be cultured from the respiratory tract samples after 8–9 days of the infection onset (Murphy, 2020). Prolonged incubation period, prolonged viral shedding in stool, and instances of recurrent infection have also been reported (Jiang et al., 2020; Landi et al., 2020; Salzberger et al., 2020; Walsh et al., 2020).

This flu-like illness began in December 2019 and by January 30, 2020 spread to 18 countries, which prompted the World Health Organization to declare it as a public health emergency of international concern (PHEIC)^1^. The first reported cases of COVID-19 were traced to the Huanan seafood market in Wuhan City, in the Hubei Province in China, in December 2019 (Zhu et al., 2020). At the time of writing the manuscript, over 280,119,931 confirmed cases and 5,403,662 deaths have already occurred due to COVID-19 in the world, affecting more than 200 countries and their socioeconomic life^2^. The rate of transmission of the disease is higher than that of SARS-CoV and MERS-CoV (Wiersinga et al., 2020). Intensive studies addressing the epidemiology, genetics, and pathogenesis of the virus have led to the rapid development and rollout of a number of vaccines with proven efficacy of varying extent (Aleem et al., 2021). Several existing treatment modules have been recommended including repurposing of antiviral therapy, plasma therapy, antibiotics like azithromycin, teicoplanin, and anti-malarials like chloroquine and hydroxychloroquine, which were posited for treatment (Kupferschmidt and Cohen, 2020). Many new ones that were still in the developmental stage were also presented like remdesivir, favipiravir, lopinavir–ritonavir, and interferon-β (Baron et al., 2020; Cavalcanti et al., 2020; Kupferschmidt and Cohen, 2020). However, their direct effect on the virus and efficiency in improving the clinical presentation of the disease remain controversial (Kupferschmidt and Cohen, 2020). Serious doubts were raised over their toxicity and side effects (Gevers et al., 2020; Kupferschmidt and Cohen, 2020). At the same time, the virus has been found to have an exceptionally high rate of evolution evident from the emergence of various mutants that appeared within a short span of time (Wang et al., 2020). The revelation was facilitated by genome sequencing of the virus (Wang et al., 2020).

The first genome sequences were those isolated from the 3 patients associated with the seafood market (Zhu et al., 2020). This was followed by rapid sequencing of ten strains from nine other hosts associated with the seafood market (Lu et al., 2020). These were available in early 2020 (Lu et al., 2020; Zhu et al., 2020) and helped in tracking the phylogeny and the probable origin of the virus to bat, indicating bat–human transmission, but through an intermediate Malayan pangolin host (Lam et al., 2020; Liu et al., 2020; Lu et al., 2020). SARS-CoV-2 was found to have 86.9% nucleotide sequence identity to the bat SARS-like CoV, bat-SL-CoVZC45, and bat-SL-CoVZXC21 genomes (Zhu et al., 2020). It bore about 79% genetic relatedness to SARS-CoV and about 50% identity to MERS-CoV (Lu et al., 2020). Homology modeling revealed that it had a similar receptor binding domain structure like SARS-CoV (Lu et al., 2020). This implied that the virus uses ACE-2 (angiotensin-converting enzyme 2) as the receptor (Lu et al., 2020). All the strains associated with the seafood market showed 99.98% sequence identity to each other with the maximum difference of only four mutations (Lu et al., 2020). Thereafter, a number of strains from different places in the world were sequenced and revealed the emergence of mutants (Islam et al., 2020; Yadav et al., 2020). Thereafter, studies investigating infectivity, transmission rate, pathogenesis, and overall virulence of the virus were undertaken (Li et al., 2020; Wang et al., 2020). The availability of genome sequencing and homology modeling data has assisted in the rapid development of a number of effective vaccines like COVAXIN™ (https://www.bharatbiotech.com/covaxin.html), mRNA-1273 (Baden et al., 2021), ChAdOx1 nCoV-19 AZD1222 (Ramasamy et al., 2021), Pfizer-BioNTech COVID-19 (BNT162b2) (Oliver et al., 2020), and others among 259 vaccines, which are being produced and marketed globally (Haidere et al., 2021). These have been introduced for mass immunization in most of the countries of the world^3^. The emergence of variants has also raised concern regarding the inefficiency of serum immunoglobulins from previous infection and convalescent patients in neutralizing the virus on reinfection (Li et al., 2020). It has raised trepidations about the usefulness of the vaccines against new strains and also regarding the long-term efficacy of currently available vaccines just introduced on a mass scale (Li et al., 2020; Weisblum et al., 2020; Haidere et al., 2021). Next-generation sequencing (NGS) and the availability of highly advanced epidemiological monitoring tools have helped in tracking the spread of the disease with new-age tactics (Massacci et al., 2020). At the same time, it has also helped in understanding the dysbiosis of microbiome associated with COVID-19. The microbiome is an important factor crucial for homeostasis and healthy state of the body. Its dysbiosis has been found to be involved in the pathogenesis of several diseases. Therefore, studying the microbiome in COVID-19 will illuminate unexplored alternate avenues for understanding pathogenesis and outcome and in turn may help to identify potential therapeutic markers. In the review, we present a brief perspective on how the emergence of variants has challenged the different prophylactic and therapeutic measures currently implemented to control the virus and also complete up-to-date information on the recent analysis of microbiome in COVID-19 patients. The review has focused on the dysbiosis observed and proposed how the microbiome may be contributing toward the onset of cytokine storm, inflammation, and overall pathogenesis of COVID-19 by taking cues from other diseases. In the prevailing situation where variants may challenge the currently available prophylactic and therapeutic measures, targeting the microbiome may be a beneficial alternate avenue for COVID-19 prevention and therapy.

## First Genome Sequences of SARS-CoV-2

The genetic material of the virus is positively coiled single-stranded RNA (Laamarti et al., 2020). The SARS-CoV-2 genome is 29.8–29.9 kb in size (Elrashdy et al., 2020; Laamarti et al., 2020). The genomic organization is typical of coronaviruses (Elrashdy et al., 2020; Laamarti et al., 2020). The polyprotein ORF1ab, which is also known as the polyprotein replicase, encompasses over two-thirds of the genome at the 5’-end (Elrashdy et al., 2020; Laamarti et al., 2020). It comprises several nonstructural proteins (NSPs), which are involved in viral replication (Elrashdy et al., 2020; Laamarti et al., 2020) like the overlapping polyproteins pp1a and pp1ab (Elrashdy et al., 2020; Laamarti et al., 2020). These are required for viral replication and transcription (Elrashdy et al., 2020; Laamarti et al., 2020). Four structural proteins, namely, the spike glycoprotein, an envelope protein, a membrane protein, and nucleocapsid protein, are also encoded by the genome (Elrashdy et al., 2020; Laamarti et al., 2020). Accessory proteins ORF3a, ORF6, ORF7a, ORF7b, ORF8, and ORF10 are hypothetical proteins with unidentified functions, which are also found in the genome (Elrashdy et al., 2020; Laamarti et al., 2020).

The first genome sequences of SARS-CoV-2 were reported by Zhu et al. in early 2020 (Zhu et al., 2020). The three strains that were sequenced were isolated from three reported cases of COVID-19 identified in patients with pneumonia in Wuhan (Zhu et al., 2020). High-throughput sequencing was performed using a combination of Illumina and Nanopore platforms on RNA extracted from BALF and culture supernatant and genome sequences were obtained (Zhu et al., 2020). The contigs generated matched with lineage B of the genus β-coronavirus and showed more than 86.9% identity with SARS-CoV obtained from bat (bat-SL-CoVZC45, MG772933.1) (Zhu et al., 2020). The three sequenced genomes grouped within the sarbecovirus subgenus (Zhu et al., 2020). The three SARS-CoV-2 from Wuhan and two bat SARS-like CoV (ZC45 and ZXC21) formed a distinct clade, while human SARS-CoV (SARS coronavirus) and genetically similar SARS-like CoV from bats from southwestern China formed another clade within sarbecovirus (Zhu et al., 2020). The sequence homology of ORF 1ab (conserved replicase domains) was found to be less than 90% between SARS-CoV-2 and other β-coronaviruses leading to the conclusion that SARS-CoV2 was a novel β-coronavirus under sarbecovirus in the family Coronaviridae (Zhu et al., 2020).

Lu et al. reported ten genomic sequences of the novel coronavirus isolated from 9 inpatients from 3 hospitals in Wuhan and admitted due to viral pneumonia of unknown cause and diagnosed negative for other common respiratory pathogens (Lu et al., 2020). Sequencing was performed using Illumina and Nanopore systems generating 8 complete and 2 partial genome sequences (Lu et al., 2020). Bat-SL-CoVZC45 was used as the reference genome (Lu et al., 2020). The eight complete genome sequences were almost identical sharing 99.98% sequence identity among themselves, indicating a very recent introduction into humans (Lu et al., 2020). The maximum difference obtained was that of only 4 mutations (Lu et al., 2020). With Blastn the complete genomes showed 87.99% and 87.23% sequence identity with Bat-SL-CoVZC45 and Bat-SL-CoVZXC21, respectively (Lu et al., 2020). Sequence homology of greater than 90% compared to these two bat-derived SARS-like β-coronaviruses was observed in five regions, namely, E, M, 7, N, and 14 culminating in 98.7% sequence identity in the E gene (Lu et al., 2020). Ia and 1b showed about 90% and about 86% sequence identity, respectively (Lu et al., 2020). The S gene had the lowest sequence identity of 75% (Lu et al., 2020). Encoded protein sequences bore high identity except the spike protein and protein 13, which showed only about 80% and 73.2% sequence homology, respectively (Lu et al., 2020). The novel coronavirus was found to have about 79% similarity with SARS-CoV and about 50% with MERS-CoV (Lu et al., 2020). Homology modeling showed that the virus has an identical genomic organization like the bat-derived SARS-like β-coronaviruses (Bat-SL-CoVZC45 and Bat-SL-CoVZXC21) and SARS-CoV with only minor deletions and insertions being noted in the 12 coding regions that were identified (Lu et al., 2020). The 12 coding regions also included 1ab, S, 3, E, M, 7, 8, 9, 10b, N, 13, and 14 (Lu et al., 2020). However, SARS-CoV-2 encodes a longer spike protein compared to that of the bat SARS-like β-coronaviruses, SARS-CoV, and MERS-CoV (Lu et al., 2020). The 10 SARS-CoV-2 strains along with the two bat reference strains formed a distinct clade (clade 2) under the sarbecovirus subgenus, while SARS-CoV formed clade 3 based on WGS (Lu et al., 2020). According to phylogenetic analysis based on the complete sequence of the RNA-dependent RNA polymerase (RdRp) gene, SARC-CoV-2 and SARS-CoV were two separate and distinct clades in the phylogenetic tree (Lu et al., 2020). SARS-CoV-2 clustered with Bat-SL-CoVZC45 and Bat-SL-CoVZXC21 in the phylogenetic tree based on 1a and spike protein gene sequences, while they were distinctly segregated by sequence of the 1b gene (Lu et al., 2020).

## Emergence of Genetic Variants, Their Spatiotemporal Distribution, and Implications for COVID-19

Rapid analysis of genome sequences of many SARS-CoV-2 strains were undertaken worldwide (Koyama et al., 2020; Weber et al., 2020). It helped to detect mutations that occurred in the strains, track the emergence of new variants, and also understand the distribution of the different variants (Koyama et al., 2020; Weber et al., 2020). This analysis also showed that from time to time different genetic variants emerged and spread to different countries of the world and were soon overtaken by latter variants (Koyama et al., 2020). Different studies reporting about the emergence of variants, their genetic diversity, and spatiotemporal distribution have been presented in **Table 1**. Among these, the D614G clade was the most common and was first found in late January 2020 in China, according to a study conducted by Koyama et al. (2020). It became the largest clade in three months (Koyama et al., 2020). Earliest samples from the USA appeared to have been derived from China and belonged to basal or L84S clades, while subsequent infected samples associated with European clades, such as D614G/Q57H (Koyama et al., 2020).

**Table 1:** Genetic variants of SARS-CoV-2.

	Genetic variation from reference genome	Common Variants and spatiotemporal, distribution and epidemiological significance	Reference/footnote
1.	2,969 missense mutations, 1,965 synonymous mutations, 484 mutations in the noncoding regions, The most common SNP (single nucleotide polymorphism) was the C to T nucleotide change at the 3,037^th^ position, P4715L in the ORF1ab, D614G mutation in the spike protein; 142 noncoding deletions, 100 in-frame deletions, 11 frameshift deletions; 66 noncoding insertions, two in-frame insertions	5,775 total number of variants reported. D614G found in late January 2020 in China; L84S clades and later D614G/Q57H clades in the USA	(Koyama et al., 2020)
2.	1,516 nucleotide variations; 744 amino acid substitutions; 12 deletion sites in ORF7, ORF8, spike protein, polyprotein ORF1ab (9 deletions spanning NSP1:6, NSP2:1, NSP8:1, NSP15:1), ORF10 (1 deletion), 3’-UTR (2 deletions)	Sequences till March 2020 showed frequency of mutations was the highest in European strains followed by Asian strains North American strains showed the lowest frequency; Case fatality rates were found to be higher in the temperate countries like Spain, Italy, Belgium, France, Netherlands, and England.	(Islam et al., 2020)
3.	10 hotspot mutations in >80% viral isolates worldwide	China, Europe, USA, and India. Mutations at positions 8782 and 28144 with frequencies of 29/99 in sequences from China were found outside China only in samples from USA at moderate frequencies and in samples from India with lower frequencies. The amino acid mutations were predicted to affect replication-related proteins and affect viral secondary structure, virulence, and pathogenicity	(Weber et al., 2020)
4.	716 site mutations; 39 recurrent nonsynonymous mutations including 10 hotspot mutations; mutations were in 6 genes, ORF1ab, spike protein, membrane glycoprotein, nucleocapsid phosphoprotein, ORF3a, and ORF8. The 10 hotspot mutations were D614G mutation at spike protein (43.46%), L84S at ORF8 (23.21%). The gene encoding ORF1ab had 4 mutation hotspots—S5932F of NSP14-exonuclease, M5865V of NSP13-helicase, L3606F of NSP6-transmembrane domain, and T265I of NSP2. Four hotspot mutations in ORF3a (Q57H and G251V) and nucleocapsid phosphoprotein (R203K and G204R) (Laamarti et al., 2020)	Strains circulating in early 2020. USA strains had 44% of total mutations; 24% singleton mutations were specific to the USA. China had 22% of total mutations; France had 4%, Netherlands had 2%; 26 countries showed singleton mutations. Mutations G251V in ORF3a, L84S in ORF8, and S5932F in ORF1ab were found in genomes of all countries except in Austria and in African countries. The mutations F924F, L4715L in orf1ab, D614G in spike protein, and an intergenic variant at position 241 were present in all genomes except in those from Asia. Mutations including two recurrent mutations T265I and Q57H of the ORF3a in Algerian strains were similar to those in European strains. Ten recurrent mutations were shared by European and Dutch genomes. In strains from America, 7 mutations were present in almost all genomes. All genomes from Asia shared 2 mutations at positions 28117 and 28144. Mutations at 1059, 14408, 23403, 25563 and 1397, 11083, 28674, 29742 were shared by African and Australian strains. The number of mutations accumulating in the genome of the virus was increasing with time. In 2020 February, December, and January the average number of mutations were 9.26, 10.59, and 10.34, respectively which changed to 11.34 in March. The first mutations that occurred were in the intergenic region linked to the nucleocapsid phosphoprotein and the orf8 protein. Later, T265I, D614G, and L84S hotspot mutations in orf1ab and Spike proteins arose in late February.	(Laamarti et al., 2020)
5.	Unique mutations: 11 amino acid substitutions, 2 new substitutions I692V downstream of the transmembrane protease serine 2 (TMPRSS2)/furin cleavage site and M1229I within the transmembrane domain; 4 deletions (ΔH69/V70, Y453F, I692V, and M1229I) in addition to D614G; 35 mutations in the spike protein	Lineage B.1.1.298; cluster 5 Emerged in August–September 2020 in North Jutland, Denmark Resistance to neutralization	(Lassaunière et al., 2021)^a^
	N501Y (asparagine to tyrosine substitution at position 501 in the S gene) and the 69–70del (a deletion of 6 bases coding for histidine and valine at positions 69 and 70 in the S gene) mutations.	“VUI-202012/01,” i.e., “variant under investigation”/20I/501Y.V1/VOC 202012/01 B.1.1.7 or alpha variant in the UK Later spread to 31 other countries including USA, Canada, and India Enhanced transmissibility, with a spreading rate 70% higher than that of wild-type SARS-CoV-2 Escapes neutralization by plasma	(Andreano et al., 2020; Conti et al., 2020; Wang et al., 2020; Callaway, 2021)^a,b^
6.	Mutation N501Y	Variant 501Y.V2 or 20H/501Y.V2 or B.1.351 or beta variant South Africa and first reported on December 18, 2020 in three provinces of the country and by December 30,2020 spread to four other countries. Higher viral load, increased transmissibility and resistant to neutralization	(Andreano et al., 2020)^a,b^
7.	Ten mutations in the spike protein (L18F, T20N, P26S, D138Y, R190S, H655Y, T1027I V1176, K417T, E484K, and N501Y). Three mutations (L18F, K417N, E484K) are located in the RBD	P.1 variant Emerged in Brazil in December 2020 Gamma variant or GR/501Y.V3	(Cascella et al., 2021; Rahman et al., 2021)
8.	Spike protein mutations T19R, Δ 156,Δ157-158, L452R, T478K, R158G, D614G, P681R, and D950N; K417N mutation	B.1.617.2 (delta) variant First detected in December 2020 in India and spread to other countries Till now detected in 85 countries 40–60% more transmissible than the Alpha variant (B.1.1.7) Less responsive to vaccines Reduced neutralization	(Lopez Bernal et al., 2021)^c^
9.	Key amino acid substitutions in spike protein (RBD substitutions in bold type): A67V, del69-70, T95I, del142-144, Y145D, del211, L212I, ins214EPE, G339D, S371L, S373P, S375F, K417N, N440K, G446S, S477N, T478K, E484A, Q493R, G496S, Q498R, N501Y, Y505H, T547K, D614G, H655Y, N679K, P681H, N764K, D796Y, N856K, Q954H, N969K, L981F	B.1.1.529 (omicron) First reported by South Africa on December 24, 2021 from samples from Botswana and South Africa Later detected in Europe, Americas, Asia, and Australia Reduced neutralization *501.V2 (Cele et al., 2021), B.1.617.2 (Lopez Bernal et al., 2021), have been found to show lower post-vaccine immune response in certain individuals; for B.1.1.529 it is still uncertain (Collie et al., 2021; Karim and Karim, 2021)	^d^

^a^SARS-CoV-2 Variants (2020) Disease Outbreak News. Available at: https://www.who.int/csr/don/31-december-2020-sars-cov2-variants/en/ (Accessed January 21, 2021). ^b^Emerging SARS-CoV-2 Variants. Available at: https://www.cdc.gov/coronavirus/2019-ncov/more/science-and-research/scientific-brief-emerging-varian (Accessed January 21, 2021). ^c^SARS-CoV-2 Delta (B.1.617.2) variant of concern (VOC) (2021). Available at: https://www.ecdc.europa.eu/en/publications-data/threat-assessment-emergence-and-impact-sars-cov-2-delta-variant (Accessed September 29, 2021). ^d^Science Brief Omicron (B.1.1.529) Variant. Available at: https://www.cdc.gov/coronavirus/2019-ncov/science/science-briefs/scientific-brief-omicron-variant.html#print (Accessed December 30,2021).

Islam et al. in early 2020 conducted analysis of 2,492 complete and near-complete genome sequences deposited in the GISAID database (Islam et al., 2020). These included sequences deposited till March 2020 and were from different places in the world (Islam et al., 2020). Weber et al. compared genome sequences of 570 SARS-Cov-2 isolates from China, Europe, USA, and India with the Wuhan isolate (Weber et al., 2020) and observed that 10 hotspot mutations were found in >80% viral isolates worldwide (Weber et al., 2020). Laamarti et al. collected and analyzed 3,067 genomes from 59 countries associated with cases during the first three months after the onset of the pandemic on December 24, 2019 (Laamarti et al., 2020). Subsequent geo-referencing mutation analysis established a correlation between the mutants and their geographical distribution and helped in the identification of region-specific loci as presented in **Table 1** (Laamarti et al., 2020).

Phylogeographical analyses showed that closely related strains were distributed in different countries and indicated different sources of introduction over time (Laamarti et al., 2020). Frequent mutations in genes responsible for vital functions of the virus like replication, virulence, and pathogenesis were encountered (Korber et al., 2020). These genetic drifts consequently would alter the secondary and tertiary structures and functions of proteins involved in these physiological and metabolic activities (Korber et al., 2020). They would also, consequently, affect drug action, vaccine efficacy, and also immune recognition (Korber et al., 2020). Korber et al. showed that the variant with D614G mutation in the spike protein was the most widespread variant across the globe (Korber et al., 2020). The time of its emergence has been deduced to be late January or early February 2020, and by June 2020, it became the dominant genotype circulating globally^4^. The original D614 form was being speedily replaced by the G614 variant (Korber et al., 2020). It had a higher rate of transmission and higher infectivity (Korber et al., 2020; Wang et al., 2020). It is associated with higher viral load and higher susceptibility of infection among the younger population (Volz et al., 2021).

Another variant called cluster 5 emerged in August–September, 2020, in North Jutland, Denmark (SARS-CoV-2 Variants, 2020). This variant was associated with infection of farmed mink and had unique mutations not seen in any other strains previously, and was found to infect only 12 humans^4^ ^,^ ^5^. This variant was found to show resistance to neutralization leading to decreased duration and strength of immune protection and also reduce the long-term efficacy of vaccines and prevailing therapeutics^4^.

Recently, a rapidly spreading variant “VUI-202012/01,” i.e., “variant under investigation”/20I/501Y.V1/VOC 202012/01 B.1.1.7 (CDC, 2020), has been reported in the UK (Wang et al., 2020). It has emerged from the 20B/GR clade (lineage B.1.1.7) (Wang et al., 2020) and is phylogenetically unrelated to the SARS-CoV-2 strain circulating in the UK when the new variant was identified^4^. It contains multiple mutations including 23 nucleotide substitutions (Conti et al., 2020)^4^ and also a combination of mutations that were circulating globally discretely (Wang et al., 2020). These were the N501Y (asparagine to tyrosine substitution at position 501 in the S gene) and the 69–70del (a deletion of 6 bases coding for histidine and valine at positions 69 and 70 in the S gene) mutations (Wang et al., 2020). The variant has enhanced transmissibility, with a spreading rate 70% higher than that of wild-type SARS-CoV-2 (Conti et al., 2020), and escapes neutralization by plasma (Andreano et al., 2020), although change in disease severity was not observed^4^. The variant was later found to have spread to at least 31 other countries including the USA, Canada, and India (Callaway, 2021)^5^.

Another variant, 501Y.V2 or 20H/501Y.V2 or B.1.351^5^, was found to emerge in South Africa^4^. This variant has the same mutation N501Y like the UK variant; however, the two variants are phylogenetically not related^4^. In December 2020, a new variant, named the SARS-CoV-2 Delta (B.1.617.2) variant of concern (VOC), was first detected in India (Lopez Bernal et al., 2021)^6^. It has higher transmissibility than other contemporary variants and has spread worldwide (Lopez Bernal et al., 2021)^6^. At present, this is the dominant variant across the world, particularly in Asia, America, and Europe^6^. It harbors the K417N mutation responsible for immune escape and affects the binding of the spike protein to the ACE2 receptor^6^. Lopez et al. conducted an analysis on its response to currently available vaccines and concluded that the vaccine showed only 67% effectiveness on delta variants compared to 74.5% on alpha variants, thereby raising trepidations on the success and long-term outcome of vaccination against SARS-CoV-2 (Lopez Bernal et al., 2021).

Andreano et al. examined the effect of convalescent plasma on the wild-type virus and subsequently on natural mutant strains detected by sequencing the genome on subsequent passages of the wild-type strain after 45 days (Andreano et al., 2020). Neutralization was found decreasing after that time period evident from a decrease in neutralizing titer (Andreano et al., 2020). An initial deletion of F140 (deletion of phenylalanine at position 140) in the N-terminal domain (NTD) N3 loop of spike protein in 36% virions and subsequently an E484K substitution in the receptor-binding domain (RBD) and later an insertion in the NTD N5 loop containing a new glycan sequence were observed on simultaneous passage and RNA sequencing (Andreano et al., 2020). The variant generated was completely resistant to plasma neutralization, while the wild type had been fully susceptible for 7 passages (45 days) and had bound to S-protein trimer and also S1 and S2 subunits (Andreano et al., 2020). With the aid of computational modeling, the researchers predicted that deletion and insertion in loops N3 and N5 prevented the binding of neutralizing antibodies (Andreano et al., 2020). They concluded that these mutations would confer complete resistance against neutralization by plasma and intervene with long-term protection by vaccines and natural antibodies (Andreano et al., 2020).

Pachetti et al. analyzed 220 genomes deposited in the GISAID database from different places in the world (Pachetti et al., 2020). They used Clustal Omega for genome alignment and characterized 8 novel recurrent mutations (Pachetti et al., 2020). These were observed at 1397, 2891, 14408, 17746, 1785, 18060, 23403, and 28881 positions (Pachetti et al., 2020). Mutations at positions 2891, 3036, 14408, 23403, and 28881 predominantly occurred in Europe, while mutations at positions 17746, 17857, and 18060 were present only in genomic sequences from North America (Pachetti et al., 2020). A silent mutation in the RdRp gene was reported first in England (UK) on February 9th, 2020, and subsequently, the authors detected a different mutation in RdRp on February 20th, 2020 in Italy (Lombardy) (Pachetti et al., 2020). The authors reported that viruses with RdRp mutation have a median of 3 point mutations, and for other mutations, a median of 1 mutation was found (Pachetti et al., 2020). These findings indicated that the virus was evolving very fast and that continent-specific mutations existed (Pachetti et al., 2020). Strains from North America, Europe, and Asia have different mutation patterns, although such strains have been found to coexist in many places (Pachetti et al., 2020). RdRp is the target for several drugs, and structural prediction showed the presence of a binding moiety in the RdRp hydrophobic cleft, adjacent to the 14408 mutation detected in this study (Pachetti et al., 2020). The findings led the authors to predict that the mutations might interfere with drug action and give rise to drug-resistant viral phenotypes (Pachetti et al., 2020).

Rahman et al. addressed the mutational changes taking place in E protein (Rahman et al., 2021). Although with mutational analysis they found that only 1.2% strains had undergone 115 unique amino acid substitutions indicating that 98.8% of the E protein of SARS-CoV-2 strains were highly conserved, latter analysis proved ominous (Rahman et al., 2021). About 58.77% nucleotide positions in the E gene had a total of 176 unique mutations globally (Rahman et al., 2021). Higher variations were observed in the C-terminal domain (CTD) of the E protein, particularly at Ser55-Phe56, Arg69, and the C-terminal end (DLLV: 72–75) (Rahman et al., 2021). The authors opined that this would affect the binding of E protein to tight junction-associated PALS1 and could affect COVID-19 pathogenesis (Rahman et al., 2021). The study reported about the V25A mutation in the transmembrane domain, which is an important factor for the homopentameric conformation of E protein and a triple cysteine motif harboring mutation L39M, A41S, A41V, C43F, C43R, C43S, C44Y, and N45R predicted to inhibit the binding of E protein with spike glycoprotein (Rahman et al., 2021). Similar analysis was conducted by Rahman et al. on 61,485 sequences of the N protein, the alternative vaccine target after spike protein (Rahman et al., 2020). The authors identified 1,034 unique nucleotide mutations out of which 367 were in primer binding sites of 11 primer sets (Rahman et al., 2020). A total of 684 amino acid substitutions were found at 317 unique positions including 82, 21, and 83 present in the RNA binding NTD, SR-rich region, and C-terminal dimerization domain, respectively (Rahman et al., 2020). Eleven in-frame deletions were detected in the linker region, and the remaining were within the NTD region (Rahman et al., 2020). High-frequency co-occurring mutations (R203K and G204R) contributed to decreasing structural flexibility (Rahman et al., 2020).

The studies documented above revealed that genome evolution is a common phenomenon in SARS-CoV-2. It has led to the emergence of genotypes with enhanced transmissibility and virulence as a result of genetic drift (Rahman et al., 2020). In a recent study reported by Gaebler et al., it was found that the viral mRNA and proteins persisted in the small intestinal epithelia months after infection and that B-cell memory response persists even after 6 months of first exposure to the virus and evolves with time (Gaebler et al., 2021). These findings suggest that the once exposed individual would be able to mount an immune response to the virus on reexposure (Gaebler et al., 2021). However, many authors have also speculated that the long-term protection of natural antibodies and fruitfulness of currently available preventive and therapeutic measures on SARS-CoV-2 variants may fail or prove to be less effective with time (Dearlove et al., 2020; Plante et al., 2020). Islam et al. analyzed 444 genome sequences of SARS-CoV-2 retrieved from the GISAID platform and belonging to 6 Southeast Asian countries (Islam et al., 2021). They characterized the nonsynonymous mutants circulating in the geographical region (Islam et al., 2021). From the analysis of the global mutation distribution, it was found that the majority of the mutations found in the region under consideration were also prevalent in Europe and North America (Islam et al., 2021). The co-occurrence of these mutations at a high frequency in other countries of the world revealed the routes of transmission of the disease (Islam et al., 2021). Unique spike protein and nonstructural protein mutations were also observed in a particular zone (Islam et al., 2021). The strains could be classified into 4 major groups and 3 subgroups based on the most frequent nonsynonymous (NS) mutations (Islam et al., 2021). A unique set of 4 co-evolving mutations were found at a high frequency within India, particularly (Islam et al., 2021). Group 2 strains were found to be common in European and North American strains (Islam et al., 2021). These had two co-evolving NS mutants, which differ in RdRp (P323L) and spike (S) protein (D614G) (Islam et al., 2021). The findings indicated that European and North American variants were dominating in Southeast Asia, indicated by a rise from 0% prevalence in January to 81% by May 2020 (Islam et al., 2021). The study predicted that these would pose a massive threat to Southeast Asia (Islam et al., 2021). To contain the spread and deal with the severity of the virus, a number of developments like antiviral therapy, vaccines, and identification of a number of useful drugs have occurred rapaciously (Alouane et al., 2020; Chen et al., 2021). However, newer studies have thrown a veil of uncertainty over their foolproof effectiveness over a broad range of variants (Wang et al., 2021). Therefore, these unforeseen consequences have purported the requirement of alternative avenues. In this light, microbiome-derived agents to encounter the pathogenesis of COVID-19 would be a beneficial aide to current methods of containment.

NGS has been beneficial for the investigation of the association of the microbiome with COVID-19 pathogenesis (Zuo et al., 2020a). These efforts have been prompted by the drive for understanding the complicated pathogenesis of the disease and in the hunt for alternative control measures for the virus. These could be successfully implemented alongside currently available treatment or at a juncture where current methods succumb to the force of genetic evolution. In view of the prevailing scenario discussed above, we present the most recent analysis related to the microbiome in the event of COVID-19 with the anticipation of understanding the prospective role that the microbiome would play in attenuating the disease burden of the ongoing pandemic.

## Microbiome Analysis in COVID-19 Patients

Futuristic investigation on oral, lung, brain, and gut microbiome has been undertaken by several researchers worldwide with an attempt to understand the involvement of the microbiome in COVID-19 pathogenesis (Zuo et al., 2020a). A number of studies have addressed the issue and have shown the involvement of the GI tract in the pathogenesis of COVID-19 and found a correlation between the microbiome and the clinical outcome of the disease (Zuo et al., 2020a). Accordingly, gut microbiome dysbiosis has been found to be associated with disease severity and progression (Zuo et al., 2020a; Yeoh et al., 2021). The depletion of commensals in the gut has been positively correlated with the severity of COVID-19 (Zuo et al., 2020a). This indicates not only the influence of the disease on the gut microbiome structure but also the significance of a healthy gut microbiome signature in the prevention of the disease onset (Zuo et al., 2020a). Similarly, lung microbiome analysis revealed dysbiosis in COVID-19 patients and yielded far-fetched results suggestive of significant involvement of the microbiome in the development of critical illness (Mostafa et al., 2020; Maes et al., 2021). All the studies on gut, lung, oral, and nasopharyngeal microbiota conducted so far have found that beneficial commensals are depleted while opportunistic pathogens undergo an upsurge in abundance (Bao et al., 2020; Mostafa et al., 2020; Zuo et al., 2020a; Nardelli et al., 2021; Yeoh et al., 2021). Moreover, the microbiome diversity was observed to be diminished in the event of COVID-19 as opposed to healthy individuals and non-COVID-19 subjects (Gu et al., 2020). **Table 2** presents a snapshot of the dysbiosis observed in the COVID-19–associated microbiome.

**Table 2:** A snapshot of the microbiome dysbiosis observed in COVID-19.

Site of sequencing	Positive correlation/enrichment	Negative correlation/decrease in abundance	References
Gut	*Coprobacillus sp., Clostridium ramosum, Clostridium hathewayi*	*Faecalibacterium prausnitzii, Bacteroides dorei, Bacteroides thetaiotaomicron, Bacteroides massiliensis, Bacteroides ovatus*,	(Zuo et al., 2020a)
Gut	*Candida albicans*, *Candida auris*, *Aspergillus flavus*		(Zuo et al., 2020b)
Gut	*Enterococcus sp*., members of *Enterobacteriaceae*	*Faecalibacterium prausnitzii*, *Clostridium butyricum*, *Clostridium leptum*, *Eubacterium rectale*	(Tang et al., 2020a)
Gut	*Collinsella aerofaciens*, *Collinsella tanakaei*, *Streptococcus infantis*, *Morganella morganii*,	*Parabacteroides merdae*, *Bacteroides stercoris*, *Alistipes onderdonkii*, and *Lachnospiraceae bacterium 1_1_57FAA*	(Zuo et al., 2021)
Gut		*Faecalibacterium prausnitzii*, *Eubacterium rectale*, Bifidobacteria	(Yeoh et al., 2021)
Gut	*Streptococcus, Rothia, Actinomyces, Vellionella*		Gu et al., 2020
Gut		*Penicillium, Aspergillus*,	Lv et al., 2021a
Lung	*Corynebacterium accolens*	*Propionibacteriaceae*	(Mostafa et al., 2020)
Lung	*Herpesvirade*		(Maes et al., 2021)
Lung	*Acinetobacter, Chryseobacterium, Burkholderia, Brevundimonas, Sphingobium, Enterobacteriaceae*		(Fan et al., 2020)
Lung	*Cutaneotricosporon, Issatchenkia, Wallemia, Cladosporium, Alternaria, Dipodascus, Mortierella, Aspergillus, Naganishia, Diutina*, and *Candida*		(Fan et al., 2020)
Lung	*alphaherpesvirus 1, rhinovirus B, human orthopneumovirus*		(Zhong et al., 2021)
Lung	*Burkholderiacepacia* complex (BCC), *Staphylococcus epidermidis, Mycoplasma spp.* (including *M. hominis and M. orale)*		(Zhong et al., 2021)
Nasopharyngeal		Proteobacteria, Fusobacteria; *Leptotrichia*, *Fusobacterium, Hemophilus, Fusobacterium peridonticum*	(Nardelli et al., 2021)
Oral	*Streptococcus, Porphyromonas, Abiotrophia, Enterobacter*, *Neisseria mucosa*, *Veillonella parvula*, *Lactobacillus fermentum*, *Enterococcus faecalis*, *Atopobium parvulum*, *Acinetobacter baumannii*, *Prevotella melaninogenica*, *jejuni*, *denticola*, and *oris*; *Eikenella corrodens*; *Capnocytophaga sputigena* and *gingivalis*; and *Aggregatibacter aphrophilus), Aspergillus sp., Nakaseomyces sp., and Malassezia sp., Candida sp., Saccharomyces sp.*, Epstein–Barr virus, Staphylococcus phage ROSA, Streptococcus phage EJ-1, phage PH10, Lactobacillus phage phiadh.	*Rothia*, *Fusobacterium*, *Haemophilus parainfluenzae* and *parahaemolyticus*, *Gemella morbillorum* and *sanguinis*, *Parvimonas micra*, and *Neisseria subflava*	(Soffritti et al., 2021)

### a. Gut Microbiome in COVID-19

Zuo et al., in a pilot study, investigated the dysbiosis of fecal microbiomes of patients with COVID-19 and investigated its association with disease severity (Zuo et al., 2020a). Whole-genome shotgun sequencing was performed on fecal samples from 15 patients with COVID-19 in Hong Kong (Zuo et al., 2020a). Fecal samples were collected 2 to 3 times weekly from the time of hospitalization till discharge (Zuo et al., 2020a). They classified disease severity as mild, moderate, severe, or critical depending on clinical parameters (Zuo et al., 2020a). The results were compared with those from 6 patients with community-acquired pneumonia and 15 healthy individuals (Zuo et al., 2020a). They found a distinct pattern of microbiome dysbiosis in COVID-19 patients compared with controls (Zuo et al., 2020a). It was characterized by an enrichment of opportunistic pathogens and a decrease in the abundance of beneficial commensals throughout the period of hospitalization (Zuo et al., 2020a). Symbionts were found to be depleted and gut dysbiosis persisted even after clearance of infection (Zuo et al., 2020a). Abundance of *Coprobacillus*, *Clostridium ramosum*, and *Clostridium hathewayi* correlated with severity (Zuo et al., 2020a). Abundance of *Faecalibacterium prausnitzii* and disease severity were negatively correlated (Zuo et al., 2020a). *Bacteroides dorei*, *Bacteroides thetaiotaomicron*, *Bacteroides massiliensis*, and *Bacteroides ovatus*, which have been reported to downregulate the expression of angiotensin-converting enzyme 2 (ACE2) in murine gut, was inversely correlated with viral load in feces of patients during the entire period of hospitalization (Zuo et al., 2020a).

Zuo et al. also conducted the analysis of dysbiosis of mycobiome during disease and recovery in COVID-19 patients using WGS of 30 fecal samples of hospitalized patients in Hong Kong and 30 control samples from healthy individuals and 9 cases of community-acquired pneumonia (Zuo et al., 2020b). They detected an increase in the abundance of *Candia albicans* and a highly heterogeneous mycobiome composition at the time of hospitalization (Zuo et al., 2020b). There was no significant difference between the fecal mycobiomes of 22 COVID-19 patients and those of controls during hospitalization (Zuo et al., 2020b). However, 8 COVID-19 patients showed significant difference (Zuo et al., 2020b). COVID-19 patients showed 2.5-fold and significantly higher diversity than that of controls in the last sample (Zuo et al., 2020b). The fecal mycobiota of COVID-19 patients at all time points had higher proportions of opportunistic fungal pathogens, *Candida albicans*, *Candida auris*, and *Aspergillus flavus*, compared with controls (Zuo et al., 2020b). *A. flavus* and *A. niger* were detected in fecal samples from a subset of patients with COVID-19, even after the clearance of the virus and the resolution of respiratory symptoms (Zuo et al., 2020b).

In an attempt to establish a correlation between bacterial groups and clinical indicators of pneumonia, Tang et al. analyzed the gut microbiome of 57 COVID-19 patients with severe or critical disease (Tang et al., 2020a). It was evident from the findings that dysbiosis existed in the subjects, and changes in the gut microbial composition had an association with disease severity and hematological parameters (Tang et al., 2020a). Butyrate-producing bacteria, like *F. prausnitzii*, *Clostridium butyricum*, *Clostridium leptum*, and *Eubacterium rectale*, decreased significantly (Tang et al., 2020a). On the basis of this altered composition, it was possible to differentiate critical patients from general and severe patients (Tang et al., 2020a). Common opportunistic pathogens *Enterococcus* and *Enterobacteriaceae* were found to increase, especially in critical patients with poor prognosis (Tang et al., 2020a).

Zuo et al. conducted an RNA transcriptome-based study with fecal samples from 15 hospitalized COVID-19 patients and found a correlation between the signature of microbiome and SARS-CoV-2 infectivity (Zuo et al., 2021). Fecal samples from a higher degree of infection exhibited higher abundance of *Collinsella aerofaciens*, *Collinsella tanakaei*, *Streptococcus infantis*, and *Morganella morganii* and higher expression of nucleotide biosynthesis, amino acid biosynthesis, and glycolysis (Zuo et al., 2021). Samples with low or no SARS-CoV-2 infectivity had higher abundance of short-chain fatty acid producing bacteria like *Parabacteroides merdae*, *Bacteroides stercoris*, *Alistipes onderdonkii*, and *Lachnospiraceae bacterium 1_1_57FAA* (Zuo et al., 2021).

Yeoh et al. recently conducted a two-hospital-based cohort study to understand the involvement of the GI tract microbiome in COVID-19 patients and disease outcome (Yeoh et al., 2021). The study was aimed at finding whether gut microbiome is associated with disease severity in COVID-19 and if microbiome dysbiosis resolved with the clearance of the virus (Yeoh et al., 2021). The study included blood and stool samples from 100 patients with SARS-CoV-2 infection, and serial stool samples were collected from 27 of these patients up to 30 days after viral clearance (Yeoh et al., 2021). Gut microbiome was analyzed using shotgun sequencing (Yeoh et al., 2021). Concentration of inflammatory cytokines and blood markers was measured from plasma (Yeoh et al., 2021). The authors found that the gut microbiome was significantly different between patients and controls (Yeoh et al., 2021). *F. prausnitzii*, *Eubacterium rectale*, and Bifidobacteria were depleted in patients and remained low up to 30 days after infection clearance (Yeoh et al., 2021). These commensals are known to have immunomodulatory potential (Yeoh et al., 2021). The dysbiosis correlated with disease severity and also with elevated concentrations of inflammatory cytokines and blood markers such as C-reactive protein, lactate dehydrogenase, aspartate aminotransferase, and gamma-glutamyl transferase (Yeoh et al., 2021).

Gu et al. conducted a cross-sectional study of gut microbiome dysbiosis using fecal samples of 30 COVID-19 patients, 24 human influenza A (H1N1) patients, and 30 healthy controls (Gu et al., 2020). Based on V3–V4 16S rRNA analysis, they observed a stark difference between the composition of COVID-19 associated microbiome and that of healthy controls (Gu et al., 2020). COVID-19 microbiome was characterized by low microbial diversity but higher relative abundance of opportunistic pathogens like *Streptococcus, Rothia, Actinomyces*, and *Vellionella* and lower relative abundance of beneficial bacteria compared with healthy controls (Gu et al., 2020). Five biomarkers, *Fusicatenibacter*, *Romboutsia*, *Intestinibacter*, *Actinomyces*, and *Erysipelatoclostridium*, could be precisely used to distinguish between COVID-19 and healthy control subjects (Gu et al., 2020).

Longxian et al. conducted fecal mycobiota analysis based on ITS sequencing in 67 COVID-19 patients, 35 H1N1-infected patients, and 48 matched healthy controls (Lv et al., 2021a). They used the results and correlated them with symptoms and gut microbiota (Lv et al., 2021a). They observed that depletion of *Aspergillus* and *Penicillium* was characteristic in the diseased patients (Lv et al., 2021a). In COVID-19 patients, positive correlation was found between Mucoromycota and *Fusicatenibacter*, *Aspergillus niger* and diarrhea, and *Penicillium citrinum* was negatively correlated with C-reactive protein (CRP) (Lv et al., 2021a). In H1N1 infection, the results were strikingly different and well distinguished from those of COVID-19 individuals (Lv et al., 2021a). The authors observed that the gut mycobiota dysbiosis persisted in the patients till the time of their discharge from the hospital (Lv et al., 2021a).

### b. Lung Microbiome in COVID-19

Metatranscriptomic and metagenomic sequencing was conducted by Mostafa et al. using the Oxford Nanopore platform on nasopharyngeal swab specimens from 50 patients undergoing investigation for COVID-19, and data were analyzed using the Cosmos ID bioinformatics platform (Mostafa et al., 2020). The microbiome exhibited decreased diversity, and the composition could be significantly associated with disease (Mostafa et al., 2020). Higher abundance of *Propionibacteriaceae* and depletion of *Corynebacterium accolens* were found in negative samples (Mostafa et al., 2020).

Maes et al. determined the lung microbiome composition using 16S RNA analysis in 24 BAL (bronchoalveolar lavage) samples from COVID-19 patients receiving invasive ventilation and compared the results with non-COVID-19 samples (Maes et al., 2021). Although the distribution of organisms causing VAP (ventilator-associated pneumonia) and the pulmonary microbiome was similar between the two groups, revealing similar α and β diversity, 3 cases of invasive aspergillosis were identified among COVID-19 patients only (Maes et al., 2021). Also, *Herpesvirade* was more frequent in COVID-19 patients (Maes et al., 2021).

Fan et al. reported about the composition of lung microbiome, which they investigated from FFPE lung tissue from 20 deceased COVID-19 patients from China (Fan et al., 2020). 16S rRNA sequencing based on the V3–V4 region of the 16S ribosomal subunit followed by analysis using the QIIME V1.8.0 package revealed that the most prevalent taxa were *Acinetobacter, Chryseobacterium, Burkholderia, Brevundimonas, Sphingobium*, and *Enterobacteriaceae* in all subjects (Fan et al., 2020). They carried out ITS sequencing for studying the mycobiota in these patients and found that *Cutaneotricosporon, Issatchenkia, Wallemia, Cladosporium, Alternaria, Dipodascus, Mortierella, Aspergillus, Naganishia, Diutina*, and *Candida* were the most common genera (Fan et al., 2020). The study revealed lung microbiome dysbiosis in COVID-19 (Fan et al., 2020).

Zhong et al. characterized respiratory microbiota dysbiosis in 23 (8 mild and 15 severe) hospitalized COVID-19 patients in China using sputum, nasal swab, throat swab, anal swab, and feces (Zhong et al., 2021). Ultra-deep metatranscriptomic profiling of the samples was performed (Zhong et al., 2021). Distinct microbiome signatures were observed in the severely ill patients undergoing antibiotic therapy, and other human respiratory viruses like alphaherpesvirus 1, rhinovirus B, and human orthopneumovirus were detected in 30.8% severe cases but not in mild cases (Zhong et al., 2021). *Burkholderia cepacia* complex (BCC), *Staphylococcus epidermidis*, or *Mycoplasma* spp. (including *M. hominis* and *M. orale*) were the predominant respiratory microbial taxa detected in the severely ill patients (Zhong et al., 2021).

### c. Oral Microbiome in COVID-19

During COVID-19 infection, a large number of co-infections were found to be caused due to oral pathogens (Bao et al., 2020). These included viruses, fungi, and bacteria originating from the oral cavity (Bao et al., 2020). Marouf et al. conducted a case-control-based analysis with 568 patients of COVID-19 and showed the association of periodontitis with the severity of COVID-19 (Marouf et al., 2021). Soffritti et al. analyzed the human oral microbiome (HOM) (bacteria, virus, fungi) in COVID-19 patients using mouth rinse sample and subjecting these to WGS (Soffritti et al., 2021). They observed oral dysbiosis in the patients compared to matched controls (Soffritti et al., 2021). Dysbiosis was marked with the decrease in alpha-diversity and lower species richness, higher inflammation, and disease severity (Soffritti et al., 2021). The specific pattern of dysbiosis observed is presented in **Table 2** (Soffritti et al., 2021). *Enterobacter* sp. and *Enterococcus* sp. were identified uniquely only in COVID-19 patients (Soffritti et al., 2021). The species richness of the oral mycobiome was found to increase in COVID-19 patients and so was the oral virome (Soffritti et al., 2021).

It has been found that salivary glands act as reservoirs of SARS-CoV-2 in asymptomatics (Xu et al., 2020). Periodontal pockets have also been proposed to be sites in the oral cavity that act as reservoirs of SARS-CoV-2 (Badran et al., 2020). These reports indicate the involvement of the oral microbiota in the pathogenesis of COVID-19 (Bao et al., 2020). Poor oral hygiene causes oral microbiome dysbiosis and enriches pathogenic oral bacteria (Patel and Sampson, 2020). A number of studies have revealed the close association between oral pathogens and respiratory diseases (Scannapieco et al., 2003; Gomes-Filho et al., 2020).

### d. Nasopharyngeal Microbiome in COVID-19

Microbiome analysis of nasopharyngeal swabs revealed a strong association of the nasopharyngeal microbiota and the pathogenesis of SARS-CoV-2 (Nardelli et al., 2021). The analysis showed that although 5 phyla, Proteobacteria, Firmicutes, Bacteroidetes, Fusobacteria, and Actinobacteria, were consistently present in both the control and cases, the relative abundance of Proteobacteria and Fusobacteria was significantly reduced in COVID-19 positive individuals (Nardelli et al., 2021). At the genus level, *Leptotrichia*, *Fusobacterium*, and *Hemophilus* were significantly low in cases compared to controls (Nardelli et al., 2021). At the species level, *Fusobacterium peridonticum* was significantly reduced in COVID-19 patients compared to controls (Nardelli et al., 2021). This indicated the protective role of these bacteria in SARS-CoV-2 as they have been previously shown to be involved in the sialylation of the cell surface (Yoneda et al., 2014; Nardelli et al., 2021).

## Microbiome Signature and the Pathogenesis of COVID-19

A healthy microbiome plays a significant role in protecting against diseases (Lynch and Pedersen, 2016). It is an essential component of the host and has been often considered as an organ of the human body (Baquero and Nombela, 2012). It is involved in maintaining homeostasis, metabolic, and physiological activities, helps in the breakdown of complex nutrients like complex carbohydrates, fats and fatty acids, fermentation of nondigestible dietary residues, digestion, epithelial cell proliferation and differentiation, vitamin synthesis, and absorption of metal ions, and also accords immune protection (Guarner and Malagelada, 2003). In neonates, it has been found to help in the maturation of the immune system (Gensollen et al., 2016). It has been estimated that in the human gut lumen alone, the number of microbial cells is ten times greater than the number of eukaryotic cells reflecting the richness of diversity in its structural composition (Guarner and Malagelada, 2003). Though the composition varies with different parameters like diet, geography, ethnicity, and lifestyle and personal habits, the composition is largely affected by the clinical condition of an individual (De, 2019; Kalantar-Zadeh et al., 2020; Lv et al., 2021b). A drastic alteration in the composition has been found to occur in the event of diseases (Hay and Zhu, 2014). This shift in the microbiota composition and its total function, consequentially perturbing homeostasis, is referred to as dysbiosis. This imbalance has been found to exist in almost all diseases in which microbiome analysis has been undertaken (Levy et al., 2017). The microbiota has been found to be distinctively differential between healthy and the disease state (Ma et al., 2019), and often dysbiosis is accompanied by reduction in diversity (Gu et al., 2020). Microbiome analysis has enabled the successful establishment of specific microbiome signatures associated with different diseases (Hsiao et al., 2014; De et al., 2020). Many of the organisms associated with these signatures have been developed as prognostic and diagnostic markers and probiotics (Ritchie and Romanuk, 2012; Integrative HMP (iHMP) Research Network Consortium, 2019; Temraz et al., 2019). Today, microbiome analysis is integral in the quest for a complete understanding of pathogenesis of any disease.

The clinical presentation of COVID-19 resembles that of many other inflammatory disorders in which microbiome dysbiosis has been often reported (Integrative HMP (iHMP) Research Network Consortium, 2019; Fajgenbaum and June, 2020). Taking cues from the role of the microbiome in these inflammatory diseases, which are also characterized by comparable clinical presentation, particularly with respect to the proinflammatory state and the occurrence of cytokine storm seen in COVID-19 (Fajgenbaum and June, 2020), we may anticipate the potential role that the microbiome plays in the pathogenesis of COVID-19.

Evident from microbiome analysis in COVID-19 patients, a potential link exists between the microbiome and COVID-19 (Gu et al., 2020). Several authors have also proposed a probable link between various pathological events occurring during COVID-19 pathogenesis and microbiota dysbiosis (Saleh et al., 2020; Viana et al., 2020). Saleh et al. proposed that mitochondrial oxidative stress observed during the disease leads to microbiota dysbiosis (Saleh et al., 2020). Viana et al. proposed that ACE2, the major receptor for entry of SARS-CoV-2 (Viana et al., 2020) and which also serves as a chaperone for the amino acid transporter B^0^AT1 and ACE2/B^0^AT1 complex (Viana et al., 2020), has been shown to be modulators of gut microbiome and has proposed the association of dysfunctions of ACE2 and gut microbiota dysbiosis (Viana et al., 2020). However, all these propositions are based on indirect evidence (Saleh et al., 2020). Inference has been based on observations obtained from research related to other diseases (Saleh et al., 2020). Investigation in COVID-19 is still pending. Based on microbiome analysis results in COVID-19 and correlating it with the current knowledge of pathological conditions occurring during SARS-CoV-2 pathogenesis (Contini et al., 2020), we have speculated in the following sections how the microbiome may be associated with COVID-19 pathogenesis.

### Effect of COVID-19 Pathogenesis on the Microbiome

SARS-CoV-2 infects epithelial cells and macrophages, which express the surface receptors angiotensin-converting enzyme 2 (ACE2) and TMPRSS2 (Tay et al., 2020). These cells include the airway epithelial cells, alveolar epithelial cells, vascular endothelial cells, and macrophages in the lung (Tay et al., 2020). However, gastrointestinal (GI) manifestations like diarrhea, nausea, and vomiting in COVID-19 patients (Ong et al., 2020) were suggestive of the involvement of the infection of the GI tract and also the possible role of the gut microbiome in pathogenesis (Mao et al., 2020; Villapol, 2020). Recent studies indicate that the virus can infect the GI tract leading to inflammation of digestive tissues (Jiao et al., 2021). The mature enterocytes also express ACE2 and TMPRSS4 protease (Devaux et al., 2021). The GI tract has been also found to be a reservoir and a site of replication for SARS-CoV-2 (Devaux et al., 2021).

The RBD of S protein of the virus binds to the ACE2 receptor and is finally internalized after a plethora of cellular events well reviewed by Tay et al. (Tay et al., 2020). The most prominent clinical presentation of the viral infection in COVID-19 is the onset of ARDS, low oxygen level in blood, and “cytokine storm” characterized by heightened secretion of proinflammatory cytokines as a result of dysregulation of the immune system in SARS-CoV-2–infected individuals (Tay et al., 2020). Cytokine storm and sepsis is the cause of death in 28% of the cases of fatal COVID-19 (Tay et al., 2020). Uninhibited inflammation leads to multiorgan failure like cardiac, hepatic, renal, and subsequent damage and leads to fatality (Tay et al., 2020). Fajgenbaum and June have defined cytokine storm as “life-threatening systemic inflammatory syndromes involving elevated levels of circulating cytokines and immune-cell hyperactivation that can be triggered by various therapies, pathogens, cancers, autoimmune conditions, and monogenic disorders” (Fajgenbaum and June, 2020). The same authors opine that “Although cytokine storm is easy to identify in disorders with elevated cytokine levels in the absence of pathogens, the line between a normal and a dysregulated response to a severe infection is blurry, especially considering that certain cytokines may be both helpful in controlling an infection and harmful to the host” (Fajgenbaum and June, 2020). In COVID-19, the immunopathogenesis of SARS-CoV-2 and the subsequent onset of cytokine storm and accompanying clinical conditions aptly prove this (Tay et al., 2020). In this review, we will not outline the pathophysiology of the disease. We will only enumerate the key changes that occur during the immunopathogenesis of the virus and the markers that are elevated or suppressed as a result of the pathophysiological events. We will correlate these symptoms with their effect on the microbiome based on observations documented by studies in COVID-19 or other diseases. Based on these observations, we will anticipate how the microbiome may be associated with COVID-19 pathogenesis.

Tay et al. have iterated that SARS-CoV-2 infection downregulates the expression of ACE2 in pulmonary epithelial cells, and this has been linked to acute lung injury (Tay et al., 2020). ACE2 regulates the rennin–angiotensin system (RAS) (Tay et al., 2020). The RAS or RAAS (rennin–angiotensin–aldosterone system) is a crucial regulator of systemic blood pressure and renal function and has been implicated in cardiovascular and renal disorders (Hamming et al., 2007). Hence, a loss of function of pulmonary ACE2 dysregulates the RAS, thereby affecting blood pressure and fluid/electrolyte balance and increases inflammation and vascular permeability in the airways (Tay et al., 2020). SARS-CoV-2 slows down the conversion of Ang-II, the main effector of the RAS (Burrell et al., 2004), to antioxidant and antiatherosclerotic Ang 1-7 levels (Calò et al., 2020). ACE2 was the first reported human homologue of ACE (Donoghue et al., 2000; Dalan et al., 2020) and was discovered in 2000 (Donoghue et al., 2000; Dalan et al., 2020). The gene *ACE-2* is located on chromosome Xp22 and encodes ACE-2 protein (Dalan et al., 2020). ACE-2 receptors are highly expressed on the apical surface of the airway epithelium of the lungs (alveolar Type-2 cells), and enterocytes of the small intestine, arterial and venous endothelial cells, and arterial smooth muscle cells, in the heart, kidneys, adrenal glands, pancreas, skeletal muscle, and adipose tissues (Hamming et al., 2004; Dalan et al., 2020). Lukassen et al., in their study addressing the expression and distribution of ACE2 and TMPRSS2 in cells derived from lung tissue and subsegmental bronchial branches by single nuclei and single-cell RNA sequencing, recently found that ACE2 is mainly expressed in a transient secretory cell of subsegmental bronchial branches (Lukassen et al., 2020). These cells are associated with the RHO GTPase function and viral processes (Lukassen et al., 2020). This suggests an increased susceptibility for SARS-CoV-2 infection (Lukassen et al., 2020). The ACE2 is part of the RAS that consists of the ACE-Ang-II-AT_1_R axis and the ACE-2-Ang-1-7-Mas axis (Dalan et al., 2020). Upregulation of the ACE-Ang-II-AT_1_ R axis and downregulation of the ACE-2-Ang-1-7-Mas axis occur in metabolic disorders and also with age (Dalan et al., 2020). The activation of the ACE-Ang-II-AT_1_R axis leads to proinflammatory and profibrotic effects in the respiratory system (Dalan et al., 2020) and also causes vascular dysfunction, myocardial fibrosis, nephropathy, and insulin resistance (Dalan et al., 2020). The ACE-2-Ang-1-7-Mas axis has anti-inflammatory and antifibrotic effects on the respiratory system and induces antioxidative stress (Dalan et al., 2020). It has a protective effect on the vascular function (Dalan et al., 2020). It also accords protection against myocardial fibrosis, nephropathy, pancreatitis, and insulin resistance (Dalan et al., 2020). ACE2 binds to MAS and induces vasodilation and inhibits cell growth and epithelial cell injury (Samavati and Uhal, 2020). It has antifibrotic, antithrombotic, and anti-arrhythmogenic effects (Samavati and Uhal, 2020). SARS-CoV-2 entry perturbs vascular homeostasis by infecting endothelial cells *via* ACE2 (Collie et al., 2021). Downregulation of ACE2 leads to the reduction of MAS activation leading to prothrombotic endothelial cell phenotype and increased vascular permeability finally leading to systemic endothelial dysfunction and vasculopathy mediated by host factors like IL-6, TNF, and the complement system finally leading to coagulopathy (Collie et al., 2021). ACE2 has many beneficial roles. Normal ACE2 levels are required to combat inflammatory lung disease (Jia, 2016). ACE2 helps mesenchymal stem cells (MSCs) of the human umbilical cord to heal ischemia-reperfusion-induced lung injury (Jia, 2016). Thus, ACE2 may help in the proliferation and differentiation of MSCs and may also help to improve endothelial progenitor cell function by regulating the eNOS and Nox pathways (Jia, 2016).

Upregulation of ACE2 has been seen in many diseases like lung cancer (Gottschalk et al., 2021). This may be related to the antitumorigenic response, which leads to the synthesis of Ang1-7 peptide, a growth suppressor, which slows down the growth of the tumor through *mas* receptor activation and subsequently inhibits tumor-promoting MAP kinases (Gottschalk et al., 2021). ACE2 receptors have been found to be strongly upregulated in lungs and kidneys of K18-hACE2 mice on the intranasal inoculation of Wuhan-standard SARS-CoV-2 (Gottschalk et al., 2021). On SARS-CoV-2 infection, upregulated expression of ACE2 was found in patients with comorbidities like lung cancer, chronic lung diseases, chronic obstructive lung disease, diabetes, and hypertension (Gottschalk et al., 2021). ACE2 has been linked to the pathogenesis of chronic inflammatory lung disease, acute lung injury (ALI), asthma, hypertension, chronic obstructive pulmonary disease (COPD), and pulmonary fibrosis (Jia, 2016). Gottschalk et al. demonstrated that ACE2 in lung cancer patients infected with SARS-CoV-2 was highly elevated and holds that the overt expression of ACE2 in chronic lung disease patients facilitates SARS-CoV-2 infection and susceptibility (Gottschalk et al., 2021). Overexpression of ACE2 through Ad-ACE2 infusion in COPD induced Wistar rats was shown to lead to significant attenuation of the COPD inflammatory process through the reduction of oxidative stress and the inhibition of NF-κB and p38 MAPK pathway activation (Xue et al., 2014). Therefore, ACE2, which restrains the overactivation of the RAS system, has been a therapeutic target in these diseases (Imai et al., 2005; Xue et al., 2014), and in the current scenario, it is being targeted to reduce the morbidity of SARS-CoV-2 infection (Marquez et al., 2021).

A number of factors have been held responsible for the dysregulation of the ACE2 function (Borro et al., 2020). These include environmental pollution and pathogens, which inflict insult to epithelial tissue, dietary fiber intake, and microbiome (Jia, 2016; Sodhi et al., 2019; Borro et al., 2020; Snelson et al., 2021). Borro et al. depicted the relationship of air pollution and COVID-19 and demonstrated that the influence of particulate matter aggressively influences the susceptibility to the respiratory disease and enhances its severity (Borro et al., 2020). They studied air quality and correlated it with COVID-19 epidemiological data from 110 Italian provinces using correlation analysis and evaluated the relationship between concentrations of particulate matter (PM)_2.5_ and the incidence, the mortality rate, and the case fatality risk of COVID-19 (Borro et al., 2020). They performed bioinformatic analysis of the ACE-2 DNA sequence to identify transcription factors that may be involved in response to pollutants (Borro et al., 2020). Significant positive correlations between PM_2.5_ levels and the incidence, the mortality rate, and the case fatality rate of COVID-19 were found (Borro et al., 2020). The study showed that pollution induced the overexpression of ACE-2 in human airways, and this would facilitate SARS-CoV-2 infection (Borro et al., 2020).

Sodhi et al. demonstrated the involvement of a bacterial component in perturbing ACE2 activity leading to inflammation (Sodhi et al., 2019). The authors used a *Pseudomonas aeruginosa*–induced bacterial pneumonia mouse model and showed that pulmonary ACE2 levels vary during bacterial lung infection, and the fluctuation is critical for determining the severity of bacterial pneumonia (Sodhi et al., 2019). Preexistence and persistent deficiency of active ACE2 were deduced to lead to excessive neutrophil accumulation in mouse lungs exposed to bacterial infection, resulting in hyperinflammatory response and lung damage (Sodhi et al., 2019). These observations coupled with proposals from various authors that preexisting overexpression of ACE2 promotes infection of SARS-CoV-2 suggest that microbiota may be involved in inducing the dysregulation of ACE2 activity leading to susceptibility to SARS-CoV-2 infection and cytokine storm (Sodhi et al., 2019; Gottschalk et al., 2021).

ACE2 is a key regulator of amino acid homeostasis in the intestine, innate immunity, expression of antimicrobial peptide ecology of the gut microbiome, and transmissible susceptibility to colitis (Hashimoto et al., 2012). Tryptophan regulates ACE-2-dependent changes in epithelial immunity and the gut microbiota (Hashimoto et al., 2012). Hashimoto et al. performed 16S rDNA sequencing to study the intestinal microbiome of *Ace2* mutant mice and wild-type littermates (Hashimoto et al., 2012). The luminal ileocecal microbiome of *Ace2* mutants was strikingly altered (Hashimoto et al., 2012). Distinct operational taxonomical units (OTUs) were found to be overrepresented in *Ace2* mutant mice (Hashimoto et al., 2012). Rapamycin treatment led to changes in the ileocecal gut microbiome composition in wild-type animals, and the signature closely resembled that found in untreated wild-type animals than that found in untreated *Ace2−/y* animals (Hashimoto et al., 2012). Trp+ diet and nicotinamide treatment reverted the intestinal microbiota composition of *Ace2* mutant mice to that of untreated wild-type littermates (Hashimoto et al., 2012). ACE2 is profusely expressed in the small intestine and is scarcely detected in the colon (Perlot and Penninger, 2013). In the absence of ACE2 tryptophan uptake is impaired due to the absence of the expression of the B^0^AT1 transporter system (Perlot and Penninger, 2013). Reduced tryptophan levels give rise to reduced activity of the mTOR pathway in the small intestine, which leads to impaired expression of antimicrobial peptides from small intestinal Paneth cells (Perlot and Penninger, 2013). This, in turn, affects the composition of the intestinal microbiota (Perlot and Penninger, 2013).

Oliveira et al. evaluated changes in the small intestinal morphology and microbiota composition in MasR knockout C57BL/6 mice (Oliveira et al., 2020). The morphological changes involved an increase the in intestinal mucosa length, an increase in intestinal villi, reduction in the Lieberkühn crypt depth, an increase in the expression of cell proliferation markers Ki-67 and Cyclin D1, and an increase in TLR4, PI3K, and AKT expressions (Oliveira et al., 2020). Bacteroidetes was observed to be higher than Firmicutes (Oliveira et al., 2020). The authors proposed that due to MasR deletion changes in intestinal microbiota occurred, perhaps due to lower absorption of neutral amino acids accompanied by a consequent increase in the intestinal villi length associated with dysbiosis and LPS overproduction that finally led to cellular proliferation and cellular inflammation (Oliveira et al., 2020). These studies clearly demonstrate that ACE2 dysregulation affects the microbiome (Oliveira et al., 2020).

Downregulation of ACE2 due to SARS-CoV-2 entry leads to decreased activation of mTOR with increased autophagy leading to intestinal dysbiosis and, consequently, diarrhea (de Oliveira et al., 2020). According to the authors, SARS-CoV-2 causes a change in the intestinal microbiota leading to diarrhea through the ACE2/mTOR/autophagy pathway (de Oliveira et al., 2020).

**Figure 1:**
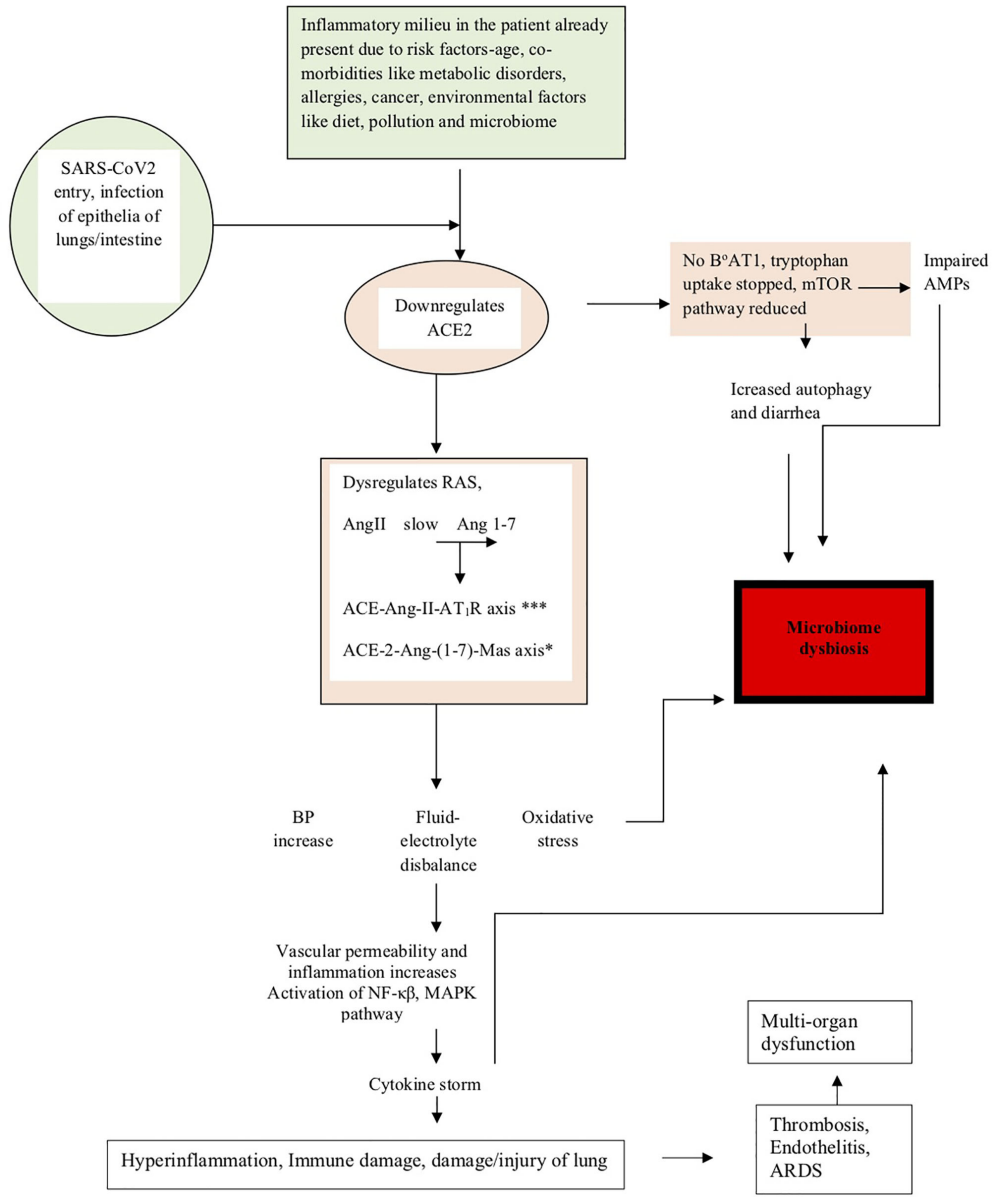
The figure shows how the pathogenesis of SARS-CoV-2 affects the microbiome. The diagram shows that in people already predisposed toward the disease due to age, comorbidities, and environmental factors like pollution, diet, and microbiome, the pathogenesis is facilitated due to prior downregulation of the ACE2 receptor, which is the major receptor for SARS-CoV-2 entry into the lung epithelia. On viral entry, ACE2 is further downregulated and initiates a cascade of events like dysregulation of RAS, slows conversion of AngII to Ang 1-7, upregulation of the ACE-Ang-II-AT_1_R axis (indicated by *** in the figure) and downregulation of the ACE-2-Ang-(1-7)-Mas axis (indicated by * in the figure), leading to clinical symptoms like an increase in blood pressure, imbalance of fluids and electrolytes, oxidative stress, vascular permeability alterations, and eventually increase in inflammation leading to the cytokine storm accentuated by the activation of NF-κβ, MAPK pathway, and finally leading to lung damage. Downregulation of ACE2 also leads to the absence of the tryptophan transporter B°AT1 leading to the inhibition of tryptophan uptake, which consequently leads to the reduction of the mTOR pathway, impaired expression of antimicrobial peptides (AMPs), and increase in autophagy leading to diarrhea and dysbiosis of the microbiome. Microbiome dysbiosis in COVID-19 has been found to be characterized by the upsurge of certain taxa and decrease of others, as indicated in **Table 2**.

The above studies have shown how dysregulation of ACE-2 expression leads to microbiota changes in COVID-19 (de Oliveira et al., 2020). The events described above have been summarized in **Figure 1**, and the events leading to microbiome dysbiosis have been speculated.

### Microbiome Induces Pathogenesis

Since microbiome has been posited as one of the environmental factors that are significantly involved in the pathogenesis of inflammatory and infectious diseases (O’Dwyer et al., 2019), therefore, next, we tried to understand by taking cues from other diseases how the microbiome can contribute to inflammation in COVID-19 and may play a significant strategic role in its pathogenesis.

O’Dwyer et al. carried out a study in a mouse model and human clinical specimens of IPF (idiopathic pulmonary fibrosis) to determine the role of the lung microbiome in local alveolar inflammation and disease progression (O’Dwyer et al., 2019). They characterized the lung microbiota in BAL fluid (BALF) from 68 patients with IPF (O’Dwyer et al., 2019). Lung microbiome was analyzed using 16S rRNA gene sequencing, and the composition was correlated with alveolar inflammation, pulmonary fibrosis, and disease progression (O’Dwyer et al., 2019). Patients with IPF with progressive disease showed significantly higher bacterial burden than nonprogressors (O’Dwyer et al., 2019). Dysbiosis of the lung microbiome was associated with the progression of disease and correlated with local host inflammation (O’Dwyer et al., 2019). Lung bacterial burden could be used to predict fibrosis progression (O’Dwyer et al., 2019). The degree of dysregulation in host alveolar inflammation was examined by comparing BALF cytokines in five healthy volunteers with that of patients with IPF (O’Dwyer et al., 2019). The authors found a significant difference in alveolar cytokine concentration in BALF between healthy control and patients with IPF (O’Dwyer et al., 2019). Significant elevation in concentrations of alveolar IL-1Ra in IPF BALF compared to control and a significant decrease in the concentrations of IL-15 were observed (O’Dwyer et al., 2019). A decrease in lung bacterial diversity was significantly associated with an increase in alveolar concentrations of proinflammatory profibrotic cytokines and growth factors, including IL-1Ra, IL-1β, CXCL8, MIP-1α, G-CSF (granulocyte colony–stimulating factor), VEGF (vascular endothelial growth factor), and epidermal growth factor (EGF) (O’Dwyer et al., 2019). A positive association between alveolar IL-6 and the relative abundance of the Firmicutes phylum, and a negative association between IL-12p70 and the relative abundance of the Proteobacteria phylum were observed (O’Dwyer et al., 2019). EGF was associated with the presence of *Lachnospiraceae*, and IL-15 showed negative correlation with the presence of *Lachnospiraceae* (O’Dwyer et al., 2019). IL-1RA showed positive correlation with the presence of Veillonella; IL-1β showed positive correlation with the presence of *Lactobacillaceae* and *Prevotella* (O’Dwyer et al., 2019). The study revealed that microbiota diversity and composition was strongly associated with increased alveolar profibrotic cytokines (O’Dwyer et al., 2019). In murine models of fibrosis, lung dysbiosis was found to precede peak lung injury and was persistent (O’Dwyer et al., 2019). In germ-free animals, the absence of a microbiome played a protective role against mortality (O’Dwyer et al., 2019). Previously, Molyneaux et al. had shown that IPF characterized by an increase in bacterial burden in BAL could be associated with the decline in lung function and death (Molyneaux et al., 2014). They found that OTUs like *Haemophilus*, *Streptococcus*, *Neisseria*, and *Veillonella* spp. were enriched in cases than in the control (Molyneaux et al., 2014). Regression analyses indicated that these OTUs as well as bacterial burden associated independently with IPF (Molyneaux et al., 2014).

Another study on the gene expression profile of BAL and peripheral whole blood samples led to the identification of two gene modules that significantly associated with IPF, BAL bacterial burden, microbial OTUs, and lavage and peripheral blood neutrophilia (Molyneaux et al., 2017). A total of 1,358 transcripts were found to be differentially expressed, and on performing GO annotation, the authors found that these were enriched for host defense and stress-related functions (Molyneaux et al., 2017). These were thioredoxin, cystatin A, chemokine-like factor superfamily member 2, S100 calcium binding protein A12, retinol binding protein 7 (Molyneaux et al., 2017), host defense-related genes like NLRC4, PGLYRP1, MMP9, and DEFA4 (Molyneaux et al., 2017), and two genes encoding specific antimicrobial peptides (SLPI and CAMP) (Molyneaux et al., 2017). Many of the transcripts were associated with survival, and their longitudinal overexpression could be associated with disease progression (Molyneaux et al., 2017). Analysis of host transcriptome and microbial signatures revealed an association between host gene expression and dysbiosis (Molyneaux et al., 2017). From persistent elevation of gene expression in longitudinal follow-up, it could be speculated that bacterial communities of the lower airways possibly act as persistent stimuli for repetitive alveolar injury in IPF (Molyneaux et al., 2017). These studies have successfully demonstrated that dysbiosis of microbiome serves as a stimulatory factor for the inflammation and induction of expression of host defense and stress-related genes (Molyneaux et al., 2017).

Yadava et al. showed that the lung microbiome plays a significant role in the onset and development of chronic obstructive pulmonary disease (COPD) using a murine model of chronic lung inflammation (Yadava et al., 2016). They compared the outcome in pathogen-free (SPF) mice and mice depleted of microbiota by antibiotic treatment or in axenic mice (Yadava et al., 2016). Animals were challenged intranasally with a mixture of LPS from *Escherichia coli* O26:B6 and porcine pancreatic elastase once a week over 4 weeks, and the authors took the terminal readout 1 week after the last challenge (Yadava et al., 2016). Lung compliance and FEV/FVC parameters were monitored (Yadava et al., 2016). Mice were tracheotomized and mechanically ventilated (Yadava et al., 2016). Microbiota was depleted before the start of the experiment with antibiotics (Yadava et al., 2016). 16S rRNA gene sequencing of the V1–V2 hypervariable region was performed to investigate the microbiome composition from BALF (Yadava et al., 2016). Microbiota-enriched BALF was intranasally inoculated into mice (Yadava et al., 2016). The authors found microbiome dysbiosis to occur upon the induction of chronic pulmonary inflammation (Yadava et al., 2016). Microbiota richness and diversity were reduced in LPS/elastase-treated mice, and an increase in *Pseudomonas*, *Chryseobacterium*, and *Lactobacillus* and a reduction in *Prevotella* occurred (Yadava et al., 2016). The airways of diseased mice were found to be characterized by distinct microbiota compared to those of healthy mice (Yadava et al., 2016). The microbiota was found to enhance the production of proinflammatory IL-17A by T cells (Yadava et al., 2016). Mice depleted, or devoid, of microbiota exhibited an improvement in lung function and underwent reduction in inflammation and lymphoid neogenesis (Yadava et al., 2016). The absence of microbiota markedly reduced the production of IL-17A, whereas intranasal transfer of fluid enriched with the pulmonary microbiota isolated from diseased mice enhanced IL-17A production in the lungs of antibiotic-treated or axenic recipients (Yadava et al., 2016). In the presence of microbiota, neutralization of IL-17A diminished inflammation and restored lung function (Yadava et al., 2016). These studies firmly confirm that the microbiota is a key risk factor for the onset and progression of inflammation as it stimulates the production of proinflammatory cytokines (Yadava et al., 2016).

Several other studies have shown the critical involvement of microbiota in facilitating inflammation and laying the groundwork for different inflammatory diseases involving vital organs (Wang et al., 2019). Wang et al. successfully depicted how microbiota can influence the induction of liver inflammation (Wang et al., 2019). They treated ducklings with oral gavage of Ochratoxin A (OTA), analyzed microbiota in the cecum and liver with 16S rRNA sequencing, and studied inflammation in the liver (Wang et al., 2019). Intestinal microbiota was cleared with antibiotics, and subsequent fecal microbiota transplantation (FMT) ensued (Wang et al., 2019). The authors reported that OTA treatment in ducks altered the intestinal microbiota composition and structure and induced the accumulation of LPS and inflammation in the liver (Wang et al., 2019). However, on antibiotic treatment, this was inhibited indicating that OTA-induced inflammation in the liver is mediated by microbiota (Wang et al., 2019). FMT from OTA-treated ducks induced liver inflammation in antibiotic-treated ducks (Wang et al., 2019). The lower expression of mRNA and the lower protein abundance of TJP1 and Occludin in recipient ducks, elevated levels of LPS and elevated liver inflammation in recipient ducks, including higher mRNA expression of TLR4 and TNF-α, protein abundance of TLR4, Myd88, and p-p65, the ratio of p-IKBα/IKBα, secretion of IL-1β and IL-6, and inflammatory cell infiltration occurred (Wang et al., 2019). This microbiota also enhanced the levels of LPS and TNF-α in the serum (Wang et al., 2019). Kishikawa et al. conducted a metagenome-wide association study in a Japanese population with rheumatoid arthritis (RA) to understand the role of the RA-associated microbiome in the pathogenesis of RA (Kishikawa et al., 2020). They observed high abundance of the genus *Prevotella*, a significant reduction of redox reaction-related gene (R6FCZ7) in the RA microbiome, and an enrichment of metabolic pathways like fatty acid biosynthesis and glycosaminoglycan degradation in case-control comparison (Kishikawa et al., 2020). The studies described above indicated that the microbiome plays a significant role in disease development, progression, and in inducing inflammation (Yadava et al., 2016; Molyneaux et al., 2017; Wang et al., 2019).

### Mechanism of Modulation of Inflammation by the Microbiome

There have been several speculations on the mechanisms by which microbiota mediates the inflammatory process and facilitates an inflammatory milieu (Khlevner et al., 2018). Many authors have proposed that the microbiome is a key component regulating the gut–brain axis, a bidirectional communication system between the central nervous system (CNS) and the gastrointestinal tract (GIT)/ENS (enteric nervous system) through the vagus nerve or by the production of active metabolites that influence enteric function, affect the CNS, and are carried in the blood across the blood–brain barrier (Khlevner et al., 2018). Serotonin has been proposed to be a potential link between the gut and the brain (Khlevner et al., 2018). Moreover, the gut microbiota has been found to be a modulator of serotonin biosynthesis (Reigstad et al., 2015). Numerous studies have shown a crucial link among the microbiome, serotonin production, and the CNS and ENS (O’Mahony et al., 2015).

Yaghoubfar et al. conducted a recent study to examine the role of gut microbiota members like *Akkermansia muciniphila* and *Fecalibacterium prausnitzii* on the serotonin system (Yaghoubfar et al., 2021). They examined the effect of these bacteria and their extracellular vesicles (EVs) on the gene expression of the serotonin system using Caco-2 cells (Yaghoubfar et al., 2021). The differentiated Caco-2 cells were treated with *A. muciniphila* and *F. prausnitzii.* After 24 h, the serotonin level was measured using ELISA, and the gene expression of serotonin system-related genes was studied using qPCR (Yaghoubfar et al., 2021). They found that treatment of cells with EVs increased the serotonin level, while bacteria failed to induce this effect (Yaghoubfar et al., 2021). Both bacteria significantly affected the expression of serotonin system-related genes (Yaghoubfar et al., 2021). *A. muciniphila* and *F. prausnitzii*-derived EVs also affected the expression of major genes involved in the serotonin system (Yaghoubfar et al., 2021). Lukovac et al. had previously demonstrated in a mouse ileal organoid model that these two bacteria with the help of their specific metabolites controlled epithelial gene expression (Lukovac et al., 2014). They studied the effect of SCFAs and products generated by the two commensals on the transcription of organoids (Lukovac et al., 2014). Metabolites of *A. muciniphila* were found to affect various transcription factors and genes involved in cellular lipid metabolism and growth, while products from *F. prausnitzii* had a weak effect on host transcription (Lukovac et al., 2014). *A. muciniphila* and its metabolite, propionate, modulated the expression of Fiaf, Gpr43, histone deacetylases (HDACs), and peroxisome proliferator-activated receptor gamma (Pparγ), crucial mediators of transcription factor regulation, cell cycle control, lipolysis, and satiety (Lukovac et al., 2014). A link has been deciphered between the microbiota and the kynurenine pathway, which serves as a major impetus to the development of Alzheimer’s disease (Garcez et al., 2019).

Several authors have proposed that low-grade inflammation in metabolic disorders concerning different organs like the gut, adipose tissue, skeletal muscles, liver, and brain is initiated by the microbiota *via* barrier dysfunctions (Geurts et al., 2014; Bleau et al., 2015). Recent evidence indicated that dysbiosis of the microbiome and alterations in the levels of gut peptides lead to metabolic dysregulation (Geurts et al., 2014; Bleau et al., 2015). Gut microbiome increases barrier permeability and endotoxemia and contributes to inflammation (Geurts et al., 2014). These propositions emphasize the role of the microbiome in regulating the gut–adipose tissue axis in facilitating low-grade chronic inflammation finally leading to different metabolic disorders (Geurts et al., 2014). The gut microbiome modulates the endocannabinoid system (eCB) and the apelinergic system, and dysbiosis of the microbiome leads to their dysregulation and may contribute to inflammation (Geurts et al., 2014). The eCB has been implicated also in the gut–brain axis (Geurts et al., 2014).

Remely et al. examined the role of metabolites from commensal microbiota in obesity and type 2 diabetes and found a crucial association between the composition of gut microbiota in obesity and type 2 diabetes and the epigenetic regulation of genes (Remely et al., 2014). The investigation aiming at studying the interaction of the microbiota with epigenetic regulation in the two groups compared to a lean control group was undertaken over a four-month intervention period (Remely et al., 2014). Abundance, butyryl-CoA:acetate CoA-transferase gene, and diversity were analyzed by PCR and 454 high-throughput sequencing (Remely et al., 2014). Epigenetic methylation of the promoter region of FFAR3 and LINE1 (long interspersed nuclear element 1) was analyzed (Remely et al., 2014). The diversity of the microbiota and the abundance of *F. prausnitzii* were both significantly lower in the two groups of patients compared to the control group (Remely et al., 2014). Clostridium cluster IV and Clostridium cluster XIV showed a decreasing trend in type 2 diabetics in comparison to the butyryl-CoA:acetate CoA-transferase gene (Remely et al., 2014). A higher body mass index had significant correlation with lower methylation of FFAR3 (Remely et al., 2014). Methylation of type 2 diabetics showed an increasing trend with time (Remely et al., 2014).

Trimethylamine N-oxide (TMAO) derived from the gut microbiota has been found to be associated with a high risk of developing atherosclerosis (AS) (Liu and Dai, 2020). RSV (Resveratrol), an anti-AS agent, was found to attenuate TMAO-induced AS in ApoE(-/-) mice (Chen et al., 2016). In mice, RSV decreases the levels of TMAO by inhibiting the production of trimethylamine (TMA) of commensals through gut microbiota remodeling (Liu and Dai, 2020). RSV leads to the increase in *Lactobacillus* and *Bifidobacterium*, which, in turn, raises bile salt hydrolase activity, thereby enhancing bile acid (BA) deconjugation and excretion in C57BL/6J and ApoE(-/-) mice (Chen et al., 2016). This has been found to be associated with a reduction in ileal BA (bile acid) content, repression of the enterohepatic farnesoid X receptor (FXR)–fibroblast growth factor 15 (FGF15) axis, and elevated cholesterol 7a-hydroxylase (CYP7A1) expression and hepatic BA neosynthesis (Chen et al., 2016). In the absence of microbiota, RSV neither decreases TMAO levels nor increases hepatic BA synthesis, and RSV-induced inhibition of TMAO-caused AS is also significantly abolished (Chen et al., 2016). RSV attenuates TMAO-induced AS by decreasing TMAO levels and increasing hepatic BA neosynthesis through remodeling of the gut microbiota (Chen et al., 2016). BA neosynthesis is partly regulated through the enterohepatic FXR–FGF15 axis (Chen et al., 2016). Therefore, microbiome-derived products induce inflammatory diseases, and in the absence of the microbiome, anti-inflammatory agents fail to suppress the pathogenesis of inflammatory diseases (Chen et al., 2016).

Miller et al. have proposed that curli (functional amyloid fibers produced by gram-negative enteric bacterial biofilms within the microbiota and similar to disease causing human amyloids structurally) are involved in inducing inflammation and also participate in the assembly of human amyloids (Miller et al., 2021). The effect of bacterial amyloids produced by the microbiota in the aggregation of AS (alpha-synuclein), neuronal accumulation of which occurs in neurodegenerative disorders, has been studied (Chen et al., 2016). Aged rats and transgenic *C. elegans* were exposed to amyloid protein curli of *E. coli* (Chen et al., 2016). Exposure of rats to curli-producing bacteria led to enhanced neuronal AS deposition in the gut and the brain and improved microgliosis and astrogliosis and enhanced the expression of TLR2, IL-6, and TNF in the brain unlike rats exposed to mutant bacteria incapable of curli synthesis or to vehicle alone (Chen et al., 2016). AS-expressing *C. elegans* exposed to curli-producing bacteria also showed enhanced AS aggregation (Chen et al., 2016). These results suggest that bacterial amyloid triggers the initiation of aggregation of AS and also stimulates the innate immune system (Chen et al., 2016). Tursi et al. demonstrated that curli induces a proinflammatory module consequently leading to autoimmunity (Tursi et al., 2017). They showed that DNA complexed with amyloid curli induced Toll-like receptor 9 (TLR9) activity (Tursi et al., 2017). Initially, the curli is bound by Toll-like receptor 2 (TLR2) and internalized into endosomes (Tursi et al., 2017). Subsequently, the curli–DNA immune complex binds to endosomal TLR9 and induces the production of type I IFNs (Tursi et al., 2017). In TLR2- and TLR9-mutant compared to wild-type mice, the production of anti-double-stranded DNA autoantibodies in response to curli–DNA was attenuated (Tursi et al., 2017). The study showed that amyloid curli helps to stimulate TLR9, production of type I IFNs, and production of autoantibodies (Tursi et al., 2017).

A plethora of evidence exists in favor of a crucial association between chronic bacterial infection and pathogenesis of neurodegenerative disorders like Alzheimer’s disease (Maheshwari and Eslick, 2015). This evidence has led several researchers to test the hypothesis and derive a conclusive understanding of the mechanism of involvement of the microbiome in the pathogenesis of these disorders (Maheshwari and Eslick, 2015). Szabady et al. proposed a model explaining how homeostasis is regulated at the epithelial interface of the gut with the help of two counteracting axes, the P-glycoprotein[P-gp]/endocannabinoid axis and the multidrug-resistant protein 2 [MRP2]/hepoxilin A_3_ (Szabady et al., 2018). Haran et al. conducted metagenomic analysis of stool samples of AD patients and studied P-glycoprotein (P-gp) expression in T84 cells (Haran et al., 2019). They identified clinical parameters, microbial taxa, and functional genes serving as predictors of AD dementia (Haran et al., 2019). Stool samples induced lower P-gp expression levels in case of patients without dementia or other types of dementia (Haran et al., 2019). They also identified bacterial markers for differentiating the AD microbiome and of those without dementia (Haran et al., 2019). These could be accurately associated with the loss of dysregulation of the P-gp pathway (Haran et al., 2019). The microbiome of AD patients had a lower proportion of butyrate producers and a higher proportion of OTUs associated with proinflammatory states (Haran et al., 2019). The authors concluded that the composition of the microbiome regulates homeostasis and contributes to AD pathogenesis when the abundance of proinflammatory taxa surpasses that of anti-inflammatory ones (Haran et al., 2019).

**Figure 2:**
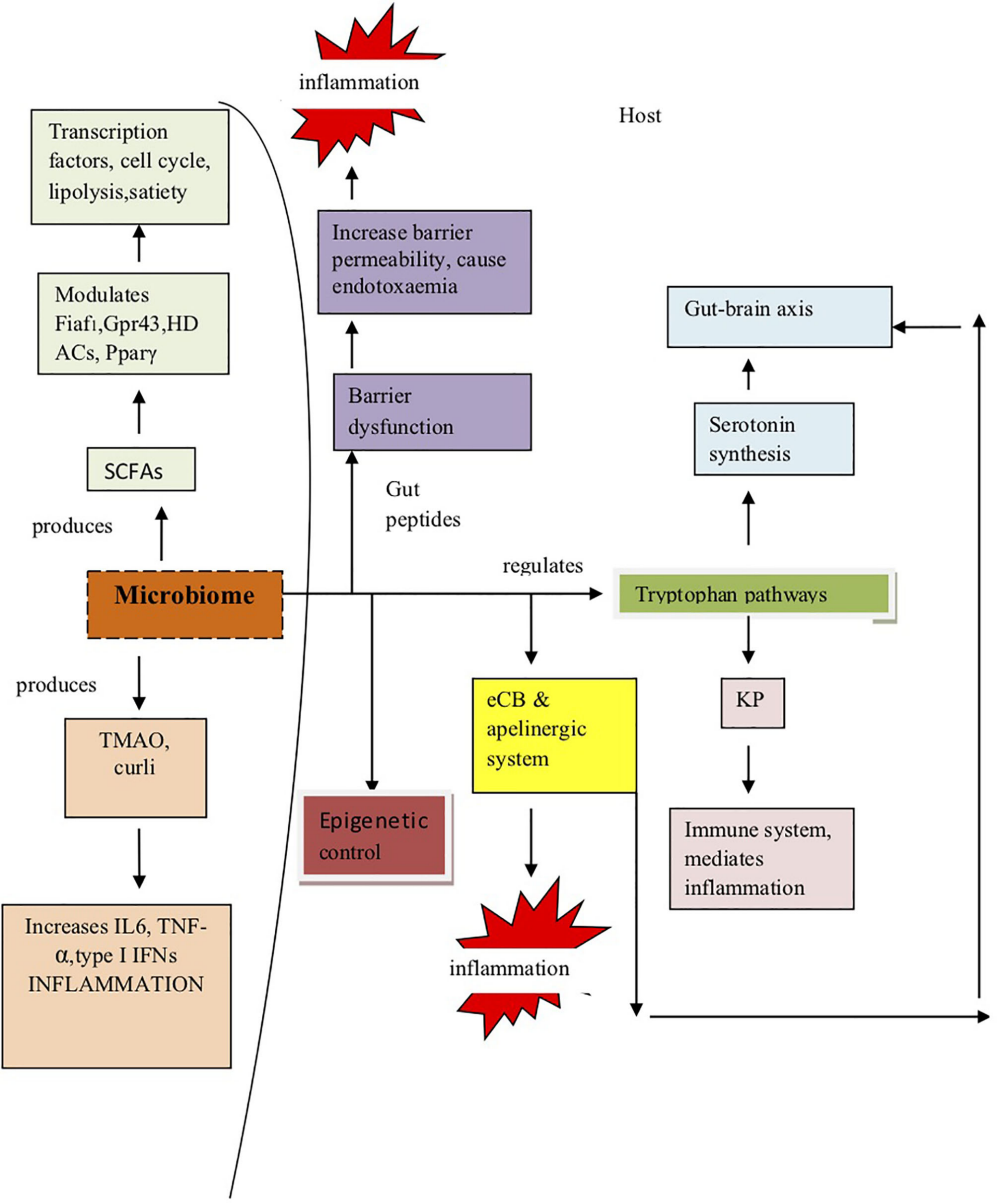
The figure shows the different mechanisms by which the microbiome has been found to regulate inflammation. The diagram shows that the microbiome undergoes dysbiosis due to various factors. The abnormal microbiome causes inflammation *via* different pathways, which may include the kynurenine pathway (KP), the endocannabinoid system (eCB), barrier dysfunction (via gut peptides), and regulation of synthesis of different host metabolites like serotonin (5-hydroxytryptamine, 5-HT). Both serotonin and kynurenine are products of tryptophan catabolism. Serotonin is a crucial neurotransmitter helping in the communication across the gut–brain axis. Apart from this, the microbiome itself produces a host of metabolites like SCFAs, TMAO, and curli, which modulates the immune system and other cellular and metabolic processes leading to outcomes like inflammation and perturbation of homeostasis. These conclusions are based on evidence gathered from other disorders. These pathways are hypothesized to significantly contribute to the pathogenesis of COVID-19, which is characterized by severe inflammation and tissue injury.

**Figure 2** summarizes the observations and speculates how microbiome dysbiosis provides a stimulus for the onset of inflammation and promotes disease development. It shows the various mechanisms by which the microbiome is speculated to regulate inflammation on the basis of evidence gathered from studies conducted in other diseases. These observations may help to hypothesize how the microbiome ignites inflammation in COVID-19.

Microbiome induces chronic inflammation in the different inflammatory diseases (Haran et al., 2019). Microbiome analysis of COVID-19 patients already described has revealed the presence of many commensals like *Parabacteroides merdae*, *Bacteroides stercoris*, *Alistipes onderdonkii*, *Lachnospiraceae bacterium 1_1_57FAA, Faecalibacterium prausnitzii* (Zuo et al., 2020a)*, Eubacterium rectale*, and *Bifidobacteria* (Yeoh et al., 2021) in the disease microbiome and a negative correlation with disease severity and pathogen load (Zuo et al., 2020a). On the other hand, several other bacteria like *Collinsella aerofaciens*, *Collinsella tanakaei*, *Streptococcus infantis*, *Morganella morganii*, *Coprobacillus*, *Clostridium ramosum*, and *Clostridium hathewayi* were found to be overrepresented in the COVID-19-associated microbiome and correlated positively with SARS-CoV-2 infectivity; these are presented in **Table 2** (Zuo et al., 2020a; Yeoh et al., 2021). The abnormal microbiome found to be associated with COVID-19 may, therefore, have a plausible role in promoting inflammation and favoring the onset of the pathogenesis of this severe disease as observed in the case of other disorders (Haran et al., 2019).

### Speculative Role of COVID-19 Microbiome

**Figure 3:**
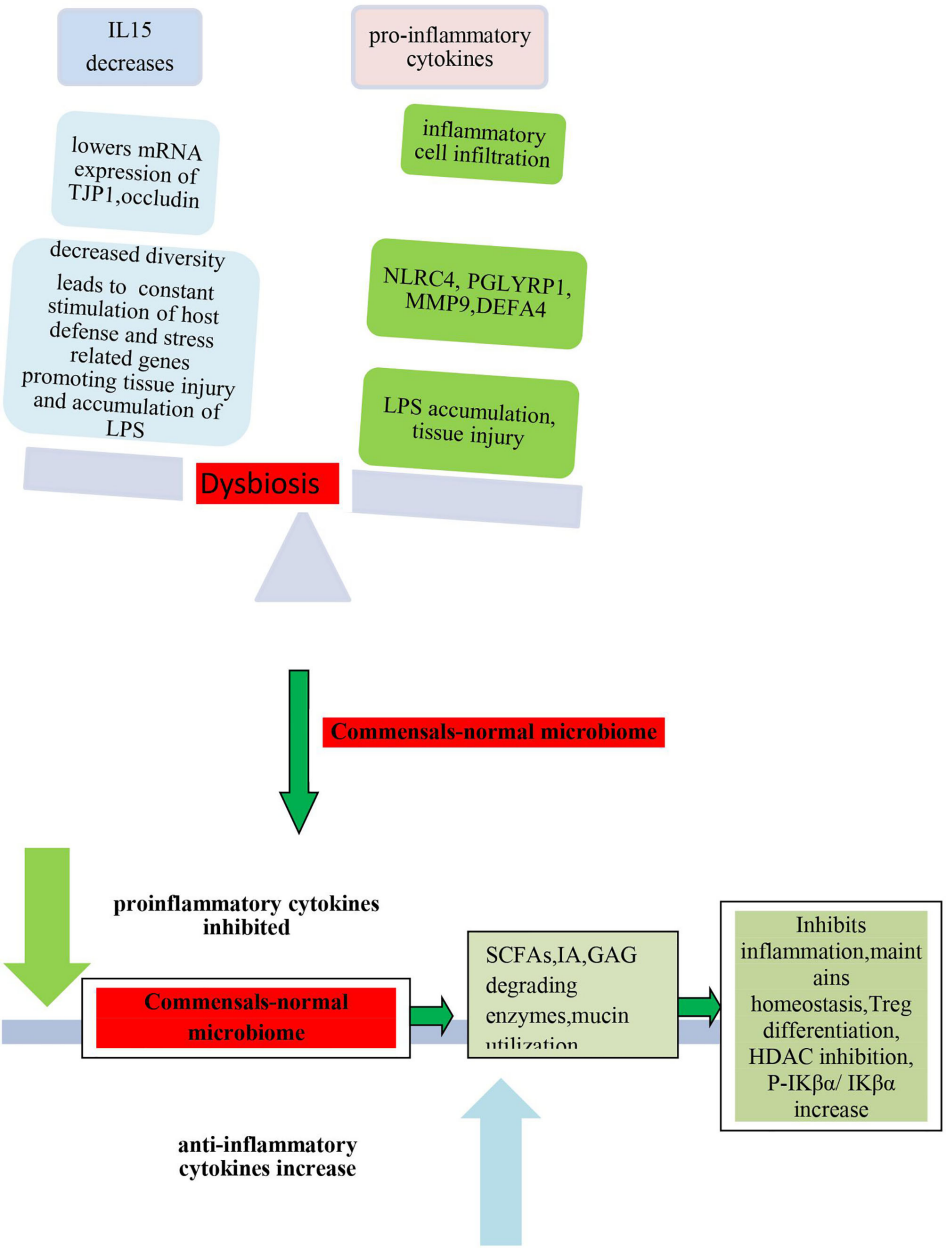
The figure provides an outline of various proinflammatory cytokines and growth factors produced as a result of dysbiosis of the microbiome indicating the crucial role that the microbiome plays in the initiation of inflammation leading to oversecretion of proinflammatory cytokines like IL6, IL-1β, CXCL8, IL-17a, IL1Ra, MIP-1α, G-CSF, VEGF, EGF, Myd88, p-p65, TLR4, and TNF-α. It shows that decreased diversity of the microbiome favors the expression of defense and stress-related host genes, which are constantly stimulated leading to tissue injury. These include NLRC4, PGLYRP1, MMP9, and DEFA4. This also promotes the accumulation of LPS in certain organs. It has been found from various studies that beneficial commensals, if present in the correct proportion, inhibit proinflammatory cytokines and promote the secretion of anti-inflammatory cytokines, promote Treg differentiation, inhibit HDACs, and increase the epithelial ratio of P-IKβα/IKβα. Indole acrylic acid (IA) produced by commensals promotes barrier function and inhibits inflammatory response. Commensals help in immunomodulation, fine-tune the balance between pro- and anti-inflammatory molecules, and regulate the immune response by regulating the differentiation of different immune cell subsets and by regulating the activity of T_reg_ cells, thereby helping in the maintenance of immune homeostasis.

Several studies have addressed the function of commensals, which have also been found to be associated with COVID-19–associated microbiome (Sokol et al., 2008). These commensals have been found to be significant for the maintenance of gut health and homeostasis, while other taxa overrepresented in the COVID-19 microbiome play an adverse role and promote an inflammatory milieu (Sokol et al., 2008). **Figure 3** shows a general outline of how microbiome dysbiosis due to increased abundance of pathobionts leads to an inflammatory state, while the presence of commensals in the correct proportion can shift this balance and suppress inflammation.

Sokol et al. evaluated the anti-inflammatory functions of *F. prausnitzii* using the Caco-2 cell line and the [2,4,6-trinitrobenzene sulfonic acid (TNBS)-induced] colitis mouse model (Sokol et al., 2008). Accordingly, in Caco-2 cells carrying a reporter gene for NF-κB activity, *F. prausnitzii* had no effect on IL-1β-induced NF-κB activity (Sokol et al., 2008). However, the supernatant was found to abolish it (Sokol et al., 2008). On the stimulation of peripheral blood mononuclear cells by *F. prausnitzii*, these produced significantly lower levels of proinflammatory cytokines IL-12 and IFN-γ and higher levels of anti-inflammatory IL-10 (Sokol et al., 2008). When live *F. prausnitzii* or its supernatant was orally administered, significant reduction in the severity of TNBS colitis occurred and reversal of the dysbiosis associated with TNBS colitis was indicated (Sokol et al., 2008). These bacteria produce metabolites able to block NF-κB activation and IL-8 production, which confer its anti-inflammatory role (Sokol et al., 2008).

The supernatant of *F. prausnitzii* has been found to regulate T helper 17 cell (Th17)/regulatory T cell (Treg) differentiation (Zhou et al., 2018). *F. prausnitzii* produces butyrate, which causes anti-inflammatory effects by inhibiting an interleukin (IL)-6/signal transducer and the activator of the transcription 3 (STAT3)/IL-17 pathway and promoting forkhead box protein P3 (Foxp3) (Zhou et al., 2018). The target of butyrate was found to be histone deacetylase 1 (HDAC1) (Zhou et al., 2018). Butyrate, from *F. prausnitzii*, maintains Th17/Treg balance and induces anti-inflammatory effects by inhibiting HDAC1 to promote Foxp3 and block the IL-6/STAT3/IL-17 downstream pathway (Zhou et al., 2018).

*F. prausnitzii* produces another metabolite, microbial anti-inflammatory molecule (MAM), that confers anti-inflammatory potential in inflammatory bowel disease (IBD) (Xu et al., 2020). MAM interacts with proteins in the tight junction pathway, including zona occludens 1 (ZO-1) (Xu et al., 2020). MAM stabilizes cell permeability and increases ZO-1 expression (Xu et al., 2020). Therefore, commensals like *F. prausnitzii* can restore the intestinal barrier structure and function mediating the regulation of the tight junction pathway and ZO-1 expression through MAM activity (Xu et al., 2020). MAM was identified by Quevrain et al., who recognized its anti-inflammatory activity and its role in inhibiting the NF-κB pathway in intestinal epithelial cells leading to prevention of colitis in an animal model (Quévrain et al., 2016).

*F. prausnetzii* and *B. thetaiotamicron* are metabolically complementary (Wrzosek et al., 2013). By modification of goblet cells and mucin glycosylation, the commensals modulate the intestinal mucus barrier (Wrzosek et al., 2013). The balance between these two commensal bacteria is essential for maintaining homeostasis in the colonic epithelia (Wrzosek et al., 2013). *B. thetaiotaomicron* is an acetate producer (Wrzosek et al., 2013). It increases goblet cell differentiation, mucus-associated gene expression, and the ratio of sialylated to sulfated mucins (Wrzosek et al., 2013). *F. prausnitzii* is an acetate consumer and a butyrate producer (1520. The synergism of the two commensals leads to diminished effects on goblet cells and mucin glycosylation (Wrzosek et al., 2013). The authors have proposed that *F. prausnitzii*, attenuates the effects of *B. thetaiotaomicron* on mucus and helps in the maintenance of appropriate proportions of different cell types of the secretory lineage in the epithelium (Wrzosek et al., 2013). It has been demonstrated in a mucus-producing cell line that acetate upregulates KLF4, a transcription factor associated with goblet cell differentiation (Wrzosek et al., 2013).

The effect of *B. thetaiotamicron* has been evaluated in Crohn’s disease (Delday et al., 2019). It has been found to improve colonic inflammation (Delday et al., 2019). *B. thetaiotaomicron* as well as its freeze-dried preparation conferred protection in both DSS and IL10 KO rodent models (Wrzosek et al., 2013). A pirin-like protein (PLP) of the bacteria reduced proinflammatory NF-κB signaling in intestinal epithelial cells (Delday et al., 2019). Recombinant PLP was found to partially recapitulate the effect of the whole strain in a rat DSS model (Delday et al., 2019). Apart from this, the bacteria have been found to have strong anti-inflammatory function as evident from its ability to regulate the intestinal immune system (Stanislav Sitkin and Juris Pokrotnieks, 2019). *B. thetaiotaomicron* by means of an anti-inflammatory PPAR-γ-dependent mechanism reduces the expression of proinflammatory cytokine through the promotion of nuclear export of the RelA subunit of NF-κB (Stanislav Sitkin and Juris Pokrotnieks, 2019). It has been found to reverse the effects of TNF-α- and IFN-γ-induced intestinal epithelial dysfunction, which leads to the modification of transepithelial resistance and permeability (Stanislav Sitkin and Juris Pokrotnieks, 2019). Extremely low levels or absence of *B. thetaiotaomicron* in feces have been found to be associated with the development of ulcerative colitis (Stanislav Sitkin and Juris Pokrotnieks, 2019), which indicates its preemptive role in protection against inflammation.

Commensals like *P. merdae* have been found to be significantly involved in regulating the gut–brain axis (Dooling and Costa-Mattioli, 2018). *P. merdae* along with another commensal, *A. muciniphila*, synergistically mediate antiseizure effects in mice (Dooling and Costa-Mattioli, 2018). They contribute to the reduction of gamma-glutamylated (GG) ketogenic amino acids (leucine, lysine, threonine, tryptophan, and tyrosine) in the colon and serum and also cause the reduction of the gamma-glutamyl transpeptidase (GGT) activity in feces (Dooling and Costa-Mattioli, 2018). These indicate that the bacteria blocking GGT activity decrease the bioavailability of the amino acids and confer protection against seizures (Dooling and Costa-Mattioli, 2018). These bacteria also increase the amounts of the major excitatory (glutamate) and inhibitory (gamma-aminobutyric acid [GABA]) neurotransmitters in the hippocampus (Dooling and Costa-Mattioli, 2018). Reduction of GABA levels or of glutamate-stimulated GABA release is associated with temporal lobe epilepsy (Dooling and Costa-Mattioli, 2018). Microbial treatment has been found to accord protection against seizures by increasing the GABA tone (Dooling and Costa-Mattioli, 2018).

Recent studies have shown the anti-inflammatory potential of several other commensals like *E. rectale* (Cattaneo et al., 2017) and *Alistipes onderdonkii* (Mobegi et al., 2020). A reduction in the abundance of *E. rectale* is associated with a peripheral inflammatory state in patients with cognitive impairment and brain amyloidosis, thereby implicating its role in brain inflammation (Cattaneo et al., 2017). The authors derived their conclusion from a study they conducted to evaluate the correlation of the abundance of proinflammatory cytokines with different taxa of the gut microbiota in brain amyloidosis in cognitively impaired patients with and without amyloidosis (Cattaneo et al., 2017). Patients with amyloidosis showed lower abundance of *E. rectale*, and negative correlation between proinflammatory cytokines IL-1β, NLRP3, and CXCL2 and abundance of *E. rectale* was observed (Cattaneo et al., 2017). Mobegi et al. conducted an interesting study in which they showed that *A. onderdonkii* could influence systolic blood pressure and modulate the risk of islanders to chronic diseases (Mobegi et al., 2020). The authors found that the presence of *A. onderdonkii* corresponded to lower systolic blood pressure, and this study revealed the anti-inflammatory role of these bacteria in the microbiome (Mobegi et al., 2020).

These beneficial bacteria whose role in inflammation was described above have been found to be depleted in the microbiome of COVID-19 patients (Zuo et al., 2020a), and this lower abundance of beneficial commensals may be partly responsible for the inflammation seen in COVID-19 characterized by an increase in IL-1β, IL-6, IFNγ, MCP1, and IP-10 (Tay et al., 2020). In more severe cases, cytokine storm occurs with higher blood plasma levels of IL-2, IL-7, IL-10, granulocyte colony-stimulating factor (G- CSF), IP-10, MCP1, macrophage inflammatory protein 1α (MIP1α), and tumor necrosis factor (TNF) (Tay et al., 2020). IL-6 levels are also elevated (Tay et al., 2020). A monocyte-derived FCN1+ macrophage population with an inflammatory function has been observed in the bronchoalveolar lavage fluid of patients with severe COVID-19 (Tay et al., 2020). A higher percentage of CD14+ CD16+ inflammatory monocytes in peripheral blood are also found in severe cases (Tay et al., 2020). These cells secrete inflammatory cytokines and chemokines including MCP1, IP-10, and MIP1α and induce the cytokine storm (Tay et al., 2020).

Recent evidence suggests that commensal microflora is indispensible for maintaining a balance between pro- and anti-inflammatory cytokines (Arpaia et al., 2013) and also modulates systemic inflammatory responses (Weaver et al., 2019). They communicate with the immune system of the host *via* their metabolites and maintain this balance (Arpaia et al., 2013). The metabolic byproducts of the commensals are sensed by the cells of the immune system (Arpaia et al., 2013). It is evident from the work of Arpaia et al. that butyrate from commensals helps in the generation of extrathymic Treg cells (Arpaia et al., 2013). An elevation in Treg cell numbers upon the addition of butyrate occurs due to the stimulation of extrathymic differentiation of Treg cells (Weaver et al., 2019). Propionate, produced by commensals and which also inhibits HDAC, was found to help in *de novo* Treg cell generation in the periphery (Arpaia et al., 2013).

Weaver et al. recently showed using a murine model that by avoiding the TLR9 tolerance and sustaining the TLR-driven immune response, the proinflammatory state can be sustained and can induce cytokine storm (Weaver et al., 2019). Mice treated with antibiotics or germ-free animals were found to respond to an initial TLR9 signal (Weaver et al., 2019). However, proinflammatory cytokine production failed on the introduction of repeated TLR9 signals *in vivo* (Weaver et al., 2019). The microbiota was found to induce JAK signaling in myeloid progenitors to facilitate TLR-enhanced myelopoiesis, which is a requirement for the accumulation of TLR-responsive monocytes (Weaver et al., 2019). When TLR-enhanced monocytopoiesis was absent, antibiotic-treated mice failed to respond to repeated TLR9 stimuli and were protected from cytokine storm–induced immunopathology (Weaver et al., 2019). There is a host of evidence suggesting the involvement of commensal microflora *via* different mechanisms in alleviating inflammation with the help of their metabolic products (Wlodarska et al., 2017).

Certain commensals like *Peptostreptococcus* sp. are capable of cleaving and transporting mucin-associated monosaccharides, thereby utilizing intestinal mucins and reducing epithelial injury (Wlodarska et al., 2017). Many *Peptostreptococcus* species containing a genetic cluster involved in tryptophan metabolite indoleacrylic acid (IA) production thereby maintaining intestinal epithelial barrier function and reducing inflammatory responses have been identified (Wlodarska et al., 2017). Several commensals like *B. sterocoris HJ-15* have been found to produce GAG-degrading enzymes like acharan sulfate lyase and heparinase (Kim et al., 1998). Others like *Enterococcus faecium*, *Lactobacillus casei*, *Lactobacillus rhamnosus*, and *Enterococcus faecalis* isolated from human fecal samples have also been found to encode GAG-degrading enzymes and carry GAG genetic clusters in their genome (Kawai et al., 2018). Many opportunistic pathogens like *Streptococcus* sp. have been found to be involved in the degradation of GAGs using their genetic clusters (Kawai et al., 2018). The cluster is involved in depolymerization, degradation, and metabolism of GAGs (Kawai et al., 2018). GAGs (glycosaminoglycans) are ubiquitously present on mammalian cell surfaces, maintain the structural integrity of the cells and tissues, serve as ligands to a plethora of signals, and participate in a host of physiological functions (Morla, 2019). They have been implicated in different types of cancer and are found to play a critical role in angiogenesis, tumor progression, and metastasis (Morla, 2019). GAGs have been associated with the inflammation of the lungs particularly during viral infections (Cheudjeu, 2020). Recent studies have demonstrated that GAGs help in the entry of the SARS-CoV-2 virus as heparin sulfate; a sulfated GAG has been found to be used as an attachment site by the virus *via* the S protein (Cheudjeu, 2020). However, earlier studies showed that degradation of GAGs like HS leads to the increase in CAMs, the endothelial cell surface adhesion molecule, and this, in turn, promotes infection (Cheudjeu, 2020). However, inhibition of heparanase, the HS degrading enzyme, leads to a decrease in lesions, inflammation, and mortality (Cheudjeu, 2020). With GAGs, a paradox is seen regarding the role of the microbiome. However, the role of commensals in the degradation of GAGs is crucial in preventing inflammation (Kawai et al., 2018).

An abundance of opportunistic pathogens in the microbiome leads to adverse reactions in inflammatory diseases as has been demonstrated in the case of colitis using a TNBS mouse model (Lee et al., 2009). This evidence suggests that the inflammatory state stimulates GAG-degrading activity of pathobionts (Lee et al., 2009). Therefore, although pathobionts with GAG-degrading activity may contribute to the aggravation of inflammatory diseases including COVID-19 (Lee et al., 2009), enzymes from the beneficial commensal flora can breakdown HS and prevent the adhesion of the virus and inhibit its entry (Cheudjeu, 2020; Martino et al., 2020). Pan et al. showed how commensals like *L. casei* may influence the pathogenesis of rheumatoid arthritis by suppressing adjuvant-induced-arthritis (AIA) significantly in a rat model (Pan et al., 2019). *L. casei* applied to AIA rats led to the inhibition of joint swelling and prevented bone destruction (Pan et al., 2019). It also reduced microbiome dysbiosis in AIA rats and downregulated the expression of proinflammatory cytokines (Pan et al., 2019). Commensals are therefore essential to maintain an anti-inflammatory environment, thereby accentuating the importance of commensals with anti-inflammatory function in the microbiome. Therefore, greater emphasis on the role of commensals in the pathogenesis of COVID-19 and prevention of the disease may be highly beneficial. The mechanism of modulation of inflammation by the microbiome may help to identify disease markers for therapy. The commensals can also be exploited as pre- and probiotics for the prevention and treatment of COVID-19. Momentous strides have been made in this direction (Hegazy et al., 2021). Hegazy et al. already found conclusive evidence that a healthy gut microbiome leads to better outcome in COVID-19 (Hegazy et al., 2021). They conducted a longitudinal study in COVID-19 patients to evaluate the role of factors like nutrients and lifestyle, which modulate gut microbiome (Hegazy et al., 2021). They found a negative correlation between the consumption of probiotic food and the severity of COVID-19 (Hegazy et al., 2021). A randomized trial is being conducted using COVID-19 patients to test the safety and effect on COVID-19 of KB109, a synthetic glycan that has been found to be formulated to modulate the gut microbiome and lead to enhancement of beneficial SCFA production in the gut (Haran et al., 2021).

## Conclusion

At present, most of the countries of the world are facing the bouts of the second wave (Pullano et al., 2021; Cele et al., 2021) due to the different variants of the SARS-CoV-2 virus (Khailany et al., 2020) like B.1.1.7 lineage *(20B/501Y.V1 variant of concern [VOC] 202012/01)* and B.1.351 lineage *(20C/501Y.V2)* identified from the UK and South Africa, respectively, followed by B.1.1.248/B1.1.28/P1 (501Y.V3) identified in Brazil and the B.1.427/B.1.429 lineage identified in California (Tang et al., 2020b; Yadav et al., 2021), and mainly the delta variant, which is at present dominant in most of the parts of the world (Rahman et al., 2021). At the time of writing the article, there have been over 278 million confirmed cases and over 5.4 million deaths worldwide^7^. A third wave is impending in these countries (Yoo, 2020; Pullano et al., 2021; Soriano et al., 2021).

When the disease started in 2019–2020, trepidations and uncertainty regarding treatment existed as scientists and the medical fraternity scrounged hard day and night to discover and deliver cure and prophylactic measures for the scourge, one of the worst in the present century (Morens et al., 2020). With time, drugs and repurposed drugs have been shown to be promising (Han et al., 2021; Braga et al., 2021). At the same time, newer revelations regarding the disease pathogenesis (Delorey et al., 2021) and the behavior of the emerging variants (Wang et al., 2021) have unfurled skepticism over the foolproof success of available prophylaxis and therapies against SARS-CoV-2 as these studies have revealed that newer variants including the recently emerged B.1.1.529 (omicron) variant show reduced response to currently available vaccines (Yoo, 2020; Cele et al., 2021; Collie et al., 2021; Haddad et al., 2021; Lopez Bernal et al., 2021).

COVID-19 has been found to introduce a host of changes in different organs in the body (Delorey et al., 2021). Transcriptional changes are also seen during the different stages of the disease and include, among others, upregulation of innate immune and inflammatory pathways (Delorey et al., 2021). These changes are accompanied by a failure of epithelial progenitor cells to regenerate eventually leading to organ failure (Delorey et al., 2021), and specific cell and gene types are prone to heritable risk (Delorey et al., 2021).

To counteract this outcome, several drugs and therapies have been recommended (Aleem et al., 2021). Some rewarding inventions include the recent engineering of IgM antibodies to produce neutralizing IgM-14 that enables them to neutralize a broad range of SARS-CoV-2 virus mutants resistant to the IgGs and provide a timely solution to the problem of antibody resistance of the virus (Ku et al., 2021). Its intranasal application has been demonstrated to be successful in rodents in conferring protection against the virus (Ku et al., 2021). Another effective innovation was the TOP1 (topoisomerase1) inhibition in suppressing the lethal inflammation induced by SARS-CoV-2 (Ho et al., 2021). Topotecan (TPT) is an FDA-approved TOP1 inhibitor and two doses of it have been found to suppress inflammation in hamsters (Ho et al., 2021). TPT treatment even after 4 days post-infection has been found to effectively reduce morbidity and mortality in a transgenic mouse model (Ho et al., 2021).

Alongside the stride made in therapeutics, the emergence of virulent variants due to frequent recombination events challenging available antidotes has occurred simultaneously (Haddad et al., 2021). Recently, 2,431 high-quality early-spread SARS-CoV-2 genome sequences from six continental groups were analyzed from GISAID (Haddad et al., 2021). The authors were able to successfully identify 1,010 unique missense mutations and seven different SARS-CoV-2 clusters (Haddad et al., 2021). Continent-specific haplotype blocks were detected (Haddad et al., 2021). Variant frequency and linkage disequilibrium was found to vary from continent to continent, particularly in North America (Haddad et al., 2021). Occurrence of recombination was evident (Haddad et al., 2021). The two most commonly occurring mutations, Spike_D614G and Nsp12_P314L, were structurally modeled, which showed that these mutations had the potential to enhance viral entry and replication, respectively (Haddad et al., 2021). It was evident that genomic recombination would lead to the enhancement of SARS-CoV-2 virulence and COVID-19 severity, which would be an obstacle for current treatment modules (Haddad et al., 2021). The remarkable findings from this study predicted that the second wave of COVID-19 would raise infection rates and mortality (Haddad et al., 2021).

Recent evidence suggests that convalescent plasma from the first-wave strains may be ineffective against the second-wave variants (Cele et al., 2021). A live-virus neutralization assay was used to compare the neutralization of a non-VOC variant with the 501Y.V2 VOC using plasma from COVID-19–infected adult patients who had been hospitalized during the two waves of the pandemic in South Africa (Cele et al., 2021). The 501Y.V2 variant was successfully neutralized by plasma from individuals who were infected during the second wave but responded ineffectively to the plasma from the first wave, although the first-wave variant was effectively neutralized by the plasma from first-wave and second-wave infections (Cele et al., 2021). These accentuate the limited efficiency of plasma therapy and its failure to provide broad-range protection (Cele et al., 2021).

501Y.V1 and 501Y.V2 variants have been found to have poor susceptibility to antibodies targeting the RBD and NTD of the spike and to sera from convalescent patients and immunized mice (Li et al., 2021; Wang et al., 2021). The neutralization resistance occurred due to E484K and N501Y mutations in the RBD of the spike (Li et al., 2021; Wang et al., 2021). Several authors have posited that variants with similar mutations in the spike protein pose new challenges for monoclonal antibody therapies and the protective efficacy of currently available vaccines (Wang et al., 2021).

In the current scenario of skepticism (Yoo, 2020), the importance of the microbiome and its involvement as a strong driving force for the onset of the pathogenesis has unfurled inadvertent insights regarding the potential mechanistic aspects, which can be exploited to derive fruitful and potential preventive and therapeutic agents.

Some alternative unconventional applications of the microbiome could be in engineering the different signaling pathways involved in inflammation and which have been found to be regulated by the microbiome (Ahmadi et al., 2020; Hoque et al., 2021), or exploiting its potency of colonization resistance that enables the microbiota to provide resistance to infection (Stacy et al., 2021). Gut microbiota from earlier infection-exposed hosts displays enhanced resistance to infection (Stacy et al., 2021). This response has been associated with altered bile acid metabolism that selects the expansion of organisms that utilize the sulfonic acid taurine (Stacy et al., 2021). Taurine accentuates the microbiota’s production of sulfide including hydrogen sulfide (Stacy et al., 2021). Sulfide is an inhibitor of cellular respiration and is, therefore, a crucial factor for host invasion by numerous pathogens that utilize host-generated oxygen for colonization (Kendall and Sperandio, 2021; Stacy et al., 2021). Blockade of sulfide would, therefore, perturb the microbiota composition and facilitate pathogen invasion (Stacy et al., 2021). There are numerous ways that could be exploited to invent new strategies of preventing infection by focusing on such mechanisms that the microbiome employs to provide protection against pathogen invasion and colonization (Stacy et al., 2021).

Another recent study showed the potential of taurine to induce autophagy, the mechanism by which the cells clear invading pathogens and alleviate infection (Wang et al., 2021). Taurine has been found to enhance PTEN activity and inhibit Akt/mTOR signaling, which reduces phosphorylation of ULK1 and ATG13 by mTOR and activates autophagy (Wang et al., 2021). Activation of autophagy accelerates the degradation of intracellular pathogens like *Streptococcus uberis* (Wang et al., 2021). This helps to reduce intracellular bacterial load and inhibition of overactivation of the NF-κB pathway, thereby reducing the inflammation and damage associated with *S. uberis* infection (Wang et al., 2021).

Ahmadi et al. demonstrated the potential of a probiotic cocktail of five *Lactobacillus* and five *Enterococcus* strains in preventing high-fat diet–induced (HFD-induced) microbiota dysbiosis, leaky gut, inflammation, metabolic dysfunctions, and degradation of physical function in older mice (Ahmadi et al., 2020). Probiotics by modulation of the microbiota could increase bile salt hydrolase activity and amounts of taurine in the gut (Ahmadi et al., 2020). This eventually stabilized tight junctions and ameliorated gut leakiness (Ahmadi et al., 2020). Taurine has been found to increase the life span of *C. elegans*, reduce adiposity and leaky gut, and enhance its physical function (Ahmadi et al., 2020). These results demonstrate the usefulness of probiotic therapies in preventing or treating aging-related leaky gut and inflammation in the elderly (Ahmadi et al., 2020).

Molecules like cytokines, metabolites, and drugs that alter epithelial tight junction (TJ) and focal adhesion morphology have been identified (Grosheva et al., 2020). The microbiome may be used to regulate the presence of these molecules in the body and inhibit inflammation. Many commensals like lactic acid bacteria (LAB) have been found to secrete exopolysaccharides (EPS), which confer protective effect against a variety of toxic compounds, stress, phage attack, and immune system, and have antimicrobial and immunomodulatory properties (Abdalla et al., 2021).

COVID-19–associated microbiome is enriched in pathogenic bacteria like *Acinetobacter*, *Sphingobium*, *Burkholderia*, and many others (Fan et al., 2020). These bacteria have been associated with proinflammatory functions (Li et al., 2019). Commensals with beneficial anti-inflammatory functions and their derivatives can inhibit these pathogenic bacteria and prevent microbiome dysbiosis (Abdalla et al., 2021). Our analysis on the role of the microbiome in the pathogenesis of COVID-19 presented in this review helped us gain insights into the diverse mechanisms in which the microbiome is significantly involved in the pathogenesis and outcome of COVID-19 and helped to conclude that, at the same time, the microbiome has the potential to prevent and treat inflammation (Abdalla et al., 2021).

Analysis of the COVID-19 microbiome showed that the microbiome is intricately associated with the pathophysiological conditions of the disease (Hoque et al., 2021). These studies have been conducted using subjects across varied populations and different age groups (Zuo et al., 2020a; Yeoh et al., 2021). The constant outcome observed irrespective of these differences was that COVID-19 presented dysbiosis of microbiome (Gu et al., 2020; Zuo et al., 2020a; Yeoh et al., 2021). The study subjects used as controls to study COVID-19–related dysbiosis reported no comorbidities, thereby presenting a profile representing dysbiosis due to COVID-19 alone (Zuo et al., 2020a; Yeoh et al., 2021). Enriching the beneficial microbes in the microbiome of the host and controlling the proportion of opportunistic pathogens (Kendall and Sperandio, 2021) will eventually help to prevent an inflammatory milieu, which could ease the onset of ARDS and other pathogenesis events in COVID-19 (Kendall and Sperandio, 2021). Lifestyle changes would be a subtle area that can help in the favorable growth of beneficial microbes and may include less exposure to pollution, abstaining from unnecessary use of antibiotics (Kendall and Sperandio, 2021), eating a diet rich in fiber, fermented foods, and prebiotics (Bousquet et al., 2021; Kendall and Sperandio, 2021) which favor the enrichment of healthy microbiota (Gasmi et al., 2021), and strict implementation of handwashing and brushing as it has been found that patients with GI symptoms suffer from worse COVID-19 outcome (Han et al., 2021; Gallardo-Escárate et al., 2021). These would help in alleviating the risk factors of inflammation and help in securing a healthy immune system, which is a prerequisite for avoiding the trauma associated with COVID-19. These insights may eventually lead to treatment and prophylactic modules for COVID-19 based on the microbiome. Another benefit of intervention based on the microbiome would be its effectiveness on all available variants of SARS-CoV-2 in contrast to current measures of prophylaxis and therapy. Therefore, the high rate of viral genomic mutation would not pose an obstacle to its long-term and broad-range efficiency. Till now, five variants designated as variants of concern (VOCs) by ECDC have been detected, namely, the Alpha (B.1.1.7), Alpha+E484K (B.1.1.7+E484K), Beta (B.1.351), Gamma (P.1), and Delta (B.1.617.2), and seven SARS-CoV-2 variants are considered variants of interest (VOIs) (https://www.ecdc.europa.eu/en/publications-data/threat-assessment-emergence-and- impact-sars-cov-2-delta-variant). This genetic diversity shows the high rate of evolution of the virus, and therefore, a pressing concern vesting upon the scientific community would be a unanimous antidote to control the infection. Microbiome could serve as a unanimous remedy for the situation.

## Author Contributions

RD conceptualized, wrote and edited the manuscript. SD edited the manuscript. All authors contributed to the article and approved the submitted version.

## Funding

The financial support of the review has been provided by the Indian Council of Medical Research, GoI, India.

## Conflict of Interest

The authors declare that the research was conducted in the absence of any commercial or financial relationships that could be construed as a potential conflict of interest.

## Publisher’s Note

All claims expressed in this article are solely those of the authors and do not necessarily represent those of their affiliated organizations, or those of the publisher, the editors and the reviewers. Any product that may be evaluated in this article, or claim that may be made by its manufacturer, is not guaranteed or endorsed by the publisher.

## Footnotes

1 Timeline WHO’s COVID-19 Response. Available at: https://www.who.int/emergencies/diseases/novel-coronavirus-2019/interactive-timeline# (Accessed January 6, 2021).

2 Coronavirus disease (COVID-19). Available at: https://covid19.who.int/ (Accessed December 29, 2021).

3 COVID-19 vaccines. Available at: https://www.who.int/emergencies/diseases/novel-coronavirus-2019/covid-19-vaccines (Accessed 16 June, 2021).

4 SARS-CoV-2 Variants (2020) Disease Outbreak News. Available at: https://www.who.int/csr/don/31-december-2020-sars-cov2-variants/en/ (Accessed January 21, 2021).

5 Emerging SARS-CoV-2 Variants. Available at: https://www.cdc.gov/coronavirus/2019-ncov/more/science-and-research/scientific-brief-emerging-varian (Accessed January 21, 2021).

6 SARS-CoV-2 Delta (B.1.617.2) variant of concern (VOC) (2021). Available at: https://www.ecdc.europa.eu/en/publications-data/threat-assessment-emergence-and-impact-sars-cov-2-delta-variant (Accessed September 29, 2021).

7 Available at: https://www.who.int/publications/m/item/weekly-epidemiological-update-on-covid-19—28-december-20212021 (Accessed December 29, 2021).
